# Assessment of Habitat Suitability and Identification of Conservation Priority Areas for Endangered Marco Polo Sheep Throughout Khunjerab National Park (Pakistan) and Tashkurgan Natural Reserve (China)

**DOI:** 10.3390/ani15131907

**Published:** 2025-06-28

**Authors:** Ishfaq Karim, Xiaodong Liu, Babar Khan, Tahir Kazmi

**Affiliations:** 1 School of Ecology and Natural Conservation, Beijing Forestry University, Haidian District, Beijing 100083, China; ishfaqkarim313@bjfu.edu.cn; 2 International Centre for Integrated Mountain Development, Kathmandu 44700, Nepal; 3 School of Forestry, Beijing Forestry University, Haidian District, Beijing 100083, China

**Keywords:** advanced modeling, climatic data, habitat suitability, Marco Polo sheep, remote sensing, transboundary conservation

## Abstract

This study assesses habitat suitability and identifies conservation priority areas for the endangered Marco Polo sheep throughout Khunjerab National Park (Pakistan) and Tashkurgan Natural Reserve (China). We analyzed species occurrence records against environmental variables (elevation, slope, climate, land cover) using MaxEnt modeling. Model performance was validated through AUC-ROC analysis and response curves, generating spatial predictions of suitable habitats to inform conservation strategies. Spatial predictions were generated to map potential distribution zones, aiding conservation planning for this endangered species. The model’s predictive performance was evaluated using the Area Under the Curve (AUC) of the Receiver Operating Characteristic curve, yielding an AUC of 0.919, indicating strong discriminatory capability. Elevation (43.9%), slope (25.9%), and September precipitation (15.9%) emerged as the most influential environmental predictors, collectively contributing 85.7% to the model. The total percentage contribution and permutation significance values were 98.6% and 77.8%, respectively. Jackknife analysis identified elevation (bio-1), slope (bio-7), hillshade (bio-2), and the maximum July temperature (bio-9) as the most significant factors influencing the distribution of Marco Polo sheep, Conversely, variables such as viewshade (bio-14), land cover (bio-3), and precipitation in August (bio-4) contributed a minimal gain, suggesting that they had little impact on accurately predicting species distribution. The habitat suitability map reveals varying conditions across the study area, with the highest suitability (yellow zones) found in the northern and western regions, particularly along the Wakhan Corridor ridgelines. The southern regions, including Khunjerab Pass, show predominantly low suitability, marked by purple zones, suggesting poor habitat conditions. The eastern region displays moderate to low suitability, with fragmented patches of green and yellow, indicating seasonal habitats. The survival of transboundary Marco Polo sheep remains at risk due to poaching activities and habitat destruction and border fence barriers. This study recommends scientific approaches to habitat restoration together with improved China–Pakistan cooperation in order to establish sustainable migratory patterns for this iconic species.

## Simple Summary

Understanding habitat suitability is critical for conserving the endangered Marco Polo sheep (*Ovis ammon polii*) across its transboundary range in Khunjerab National Park (Pakistan) and Tashkurgan Natural Reserve (China). Through MaxEnt modeling we identified crucial environmental factors at different elevation points and slope levels and monthly climate patterns to help in identifying effective conservation locations. Elevation together with slope and September precipitation proved to be critical elements in reaching an assessment model performance level of AUC = 0.919. The assessment results showed that areas within the northern and western sections of the Wakhan Corridor exhibited yellow-colored habitat quality zones with high suitability, but the Khunjerab Pass (south) and eastern sections demonstrated poor habitat conditions according to the evaluation. Based on previously documented research results, the combination of poaching threats and habitat degradation and border fences continues to endanger the survival of the species. The authors recommend that China and Pakistan develop a joint protective program to safeguard this endangered species and their habitats along their migratory pathways to sustain this emblematic ungulate.

## 1. Introduction

The Marco Polo sheep (*Ovis ammon polii*), first documented by Marco Polo in 1273, is an iconic species of the Pamir Mountains [1]. Marco Polo himself was captivated by the animal’s distinctive long, spiraling horns, which later became prized trophies for foreign hunters during the 19th century [2].

The Marco Polo sheep (*Ovis ammon polii*), a subspecies of argali, inhabits the high-altitude regions of the Pamir Mountains across Pakistan, China [1,3,4], Tajikistan [5,6], and Afghanistan [7]. Key protected areas within this range, such as China’s Taxkorgan Nature Reserve [8,9] and Pakistan’s Khunjerab National Park [10,11], support globally significant populations of wild ungulates, including the blue sheep (*Pseudois nayaur*), Marco Polo sheep (*Ovis ammon polii*), and Siberian ibex (*Capra sibirica*) [1,12]. Despite their ecological importance, comprehensive data on the abundance, distribution, and conservation status of these species remain scarce, particularly those on the Siberian ibex (*Capra sibirica*), Marco Polo sheep (*Ovis ammon polii*), and blue sheep (*Pseudois nayaur*), limiting effective habitat and population management [2].

The International Union for Conservation of Nature (IUCN) currently lists the Marco Polo sheep as “Near Threatened,” highlighting the need for ongoing conservation efforts [13]. Meanwhile, in the Karakoram and Himalayan regions, the presence of pastoral communities poses additional challenges to the sheep’s habitat [14,15]. Conservation initiatives have been proposed to address these challenges. For instance, in 2003, WWF-Pakistan suggested establishing a “Pamir International Conservancy,” and in 2004, the Aga Khan Foundation launched the “Pamir Integrated Conservation and Development Program. Additionally, the concept of a peace park has been endorsed to ensure the long-term preservation of Marco Polo sheep in this transboundary region [1].

The Marco Polo sheep (*Ovis ammon polii*) has faced significant population declines historically due to uncontrolled hunting and habitat disturbances [1,4,10,14,16]. In Pakistan, traditional hunting practices once severely impacted the species until regulatory measures were introduced by Mir Muhammad Nazim Khan of Hunza in 1892 to curb exploitation [16,17]. However, anthropogenic pressures resurged in the late 20th century with the construction of the Karakoram Highway (KKH) in the 1960s–1970s, which facilitated increased access and hunting, leading to further population declines [17]. Similar threats have been documented in China, where hunting activities within the Taxkorgan Nature Reserve (TNR) have contributed to population reductions [8]. While conservation efforts such as hunting bans in protected areas like TNR and Pakistan’s Khunjerab National Park (KNP) have been implemented, ongoing challenges hinder effective recovery [14].

MaxEnt (Maximum Entropy) modeling is widely used to assess habitat suitability and predict the distribution of endangered species [18,19,20], such as the Marco Polo sheep (*Ovis ammon polii*) [14]. This method analyzes key ecological variables influencing species occurrence and projects potential habitats under different environmental scenarios [21,22]. MaxEnt is especially valuable in regions experiencing habitat fragmentation, as it helps identify critical biodiversity zones and prioritize conservation efforts [23,24,25,26]. By integrating species occurrence data with environmental predictors (e.g., climate and topography), MaxEnt enables targeted strategies to safeguard vital habitats essential for the survival of species like the Marco Polo sheep while reducing anthropogenic pressures [14,27].

Despite their ecological importance, the habitat preferences of high-altitude ungulates like the Marco Polo sheep remain poorly understood [8]. Studies are urgently needed to assess how environmental variables influence their distribution and to identify critical habitats and their distribution in the study area [18,28].

This study investigates the distribution and habitat requirements of *Ovis ammon polii* in Khunjerab National Park (Pakistan) and Taxkorgan Nature Reserve (China) using species distribution modeling. We identify key environmental factors influencing Marco Polo sheep populations and map critical habitats across this transboundary region [20,25,29]. Our findings provide essential data for the following: (I) prioritizing conservation areas, (II) maintaining habitat connectivity, and (III) guiding China–Pakistan collaborative management strategies to protect this vulnerable high-altitude ungulate and its ecosystem [10,28,30].

## 2. Materials and Methods

### 2.1. Study Area

**Figure 1:**
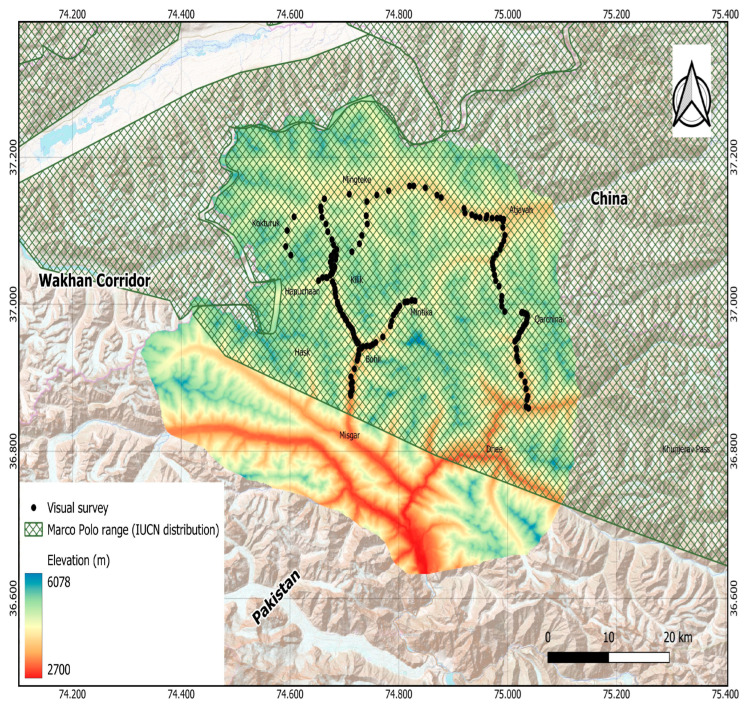
Study site map and ecosystem boundaries of Marco Polo Sheep along Pakistan–China border.

Khunjerab National Park (KNP) in Pakistan and Taxkorgan Nature Reserve (TNR) (Figure 1) in China are critical conservation areas established to protect endangered species such as the snow leopard (*Panthera uncia*) and Marco Polo sheep (*Ovis ammon polii*) [3,8,10,12,16,17,31]. KNP, located in Pakistan’s upper Hunza region between 36°01′ N and 37°02′ N and 74°55′ E and 75°57′ E, covers approximately 5544 square kilometers. Established in 1975, it is Pakistan’s third largest national park and shares a border with China’s Taxkorgan Natural Reserve [4,14,31,32].

TNR is situated at the convergence of Tajikistan, Afghanistan, China, and Pakistan, between latitudes 35°38′ and 37°30′ N and longitudes 74°30′ and 77°00′ E, encompassing an area of approximately 15,683 square kilometers [7,9,14] (Figure 1). With an average elevation exceeding 4000 m, TNR supports a unique high-mountain desert ecosystem characterized by sparse vegetation, primarily consisting of cryophytic meadows and wetland species [3,8,12]. Both protected areas are home to a considerable population of carnivores, ungulates, and rodents, including wolves (*Canis lupus*), brown bears (*Ursus arctos*), red foxes (*Vulpes vulpes*), snow leopards (*Uncia uncia*), Alpine marmots (*Marmota marmota*), blue sheep (*Pseudois nayaur*), and Siberian ibexes (*Capra sibirica*), many of which are classified as rare, vulnerable, or endangered [4,16,31].

The climate in these regions is heavily influenced by altitude, resulting in significant temperature variations. In KNP, winter temperatures can drop to −15 °C, while summer temperatures average around 14 °C. Annual precipitation is approximately 140.73 mm, with the highest rainfall occurring in May and June. In TNR, the average annual temperature is 3 °C, with precipitation trending at 75.4 mm per year [10,14]. The flora in KNP and TNR includes species such as Artemisia, Primula, Rosa, Salix, Potentilla, Populus, Hippophae, and Betula, along with perennial reed grasses, Lachnagrostis billardierei and Phleum pratense, primarily found along stream beds and flat soil areas. In contrast, TNR’s flora is subjugated by *Stipa* and other dwarf shrubs like *Artemisia* and *Ceratoides*, typical of desert steppe ecosystems [8,15,33]. The local population, primarily Wakhi, Tajik, and Brusho agro-pastoralists, relies on agriculture, livestock rearing, and seasonal tourism for their livelihoods [10,17].

A Garmin eTrex 10 handheld GPS device (Garmin Ltd., Olathe, KS, USA) was used for data collection.

### 2.2. Field Survey and Data Collection

In summer and autumn 2024, we conducted systematic habitat suitability assessments for Marco Polo sheep (*Ovis ammon polii*) across their potential range. Using standardized protocols, we collected 313 georeferenced observation points from elevated ridgeline vantage points, using a Garmin eTrex 10 handheld GPS device (Garmin Ltd, Olathe, Kansas, USA) [11,14]. These locations were selected based on prior knowledge of the species’ typical habitats. During the field survey, we employed cameras and spotting scopes (SWAROVSKI HABICHT ST 80, 80 mm) to observe wildlife [4,11]. The climate data used in this study were collected from the WorldClim version 2.1 database, which freely provides approximately 1 × 1 km^2^ spatial resolution global climate data layers.

Historical monthly weather data for 2020–2021 were sourced from WorldClim [24]. We utilized various environmental variables in our analysis, including bioclimatic factors, elevation data from a Digital Elevation Model (DEM), slope, hillshade, viewshed, and land cover datasets; however the slope, hillshade, and viewshed were derived from a 30 m DEM using ArcGIS 10.8 Spatial Analyst tools and exported in ASCII format with same cell size to apply MaxEnt modeling on the desired dataset, following the methodologies outlined by Evcin et al. [20] and Su et al. [29]. (Table 1). From the 14 WorldClim variables, the minimum temperature, maximum temperature, and precipitation were selected for their ecological relevance to species’ thermal limits and to avoid multicollinearity, ensuring reliable MaxEnt modeling. This study considered nine climatic variables, including precipitation in July, August, and September; the maximum temperature in July, August, and September; and the minimum temperature in July, August, and September [23]. Meanwhile, elevation or DEM data, which were obtained from the Advanced Spaceborne Thermal Emission and Reflection Radiometer (ASTER) with a 30 m resolution [22,27] (Table 1), were categorized into five classes at 100 m intervals for use in MaxEnt modeling, as suggested by Fatima et al. [18]. Additionally, 2022 Global Land Use/Land Cover (LULC) data from Sentinel-2, provided by ESRI at a 10 m resolution, were adjusted to a 30 m resolution and clipped to the study area boundaries for analysis, in line with procedures by Nneji et al. [22].

For Model Calibration, we converted the bioclimatic variables to ASCII file format using the SDM tool in ArcGIS 10.1, as described by Brown et al. [19] and Su et al. [29]. These environmental variables, along with species presence data, served as input parameters for the MaxEnt model to predict species distribution, following the approach of Nneji et al. [22] and Kumar and Stohlgren [24]. The MaxEnt model was run with 10 replicates using the cross-validation method to ensure model stability and minimize overfitting. For each run, 75% of the presence records were randomly allocated for training and 25% for testing. Additionally, 10,000 background points were randomly generated across the study area to improve model performance, following standard MaxEnt recommendations. The final habitat suitability map was produced by averaging the results from all replicates [27]. Model performance was assessed using the AUC of the ROC curve, where values >0.7 indicated acceptable accuracy and >0.9 high accuracy. The AUC standard deviation across replicates was used to evaluate model stability [23,34]. Response curves were generated in MaxEnt to assess how each environmental variable affects the probability of species presence, helping identify key ecological thresholds and variable influence [18].

GPS points were incorporated into a geographic coordinate system and transformed into species distribution model (SDM) input data for ArcGIS, following procedures by Evcin et al. [20] and Fatima et al. [18]. The resulting ASCII file containing all variables was used as input to run MaxEnt software (version 3.4.2) during analysis, as outlined by Su et al. [29] and Yi et al. [23].

## 3. Results

### 3.1. Average Sensitivity, Average Omission, and Jackknife Test

**Figure 2:**
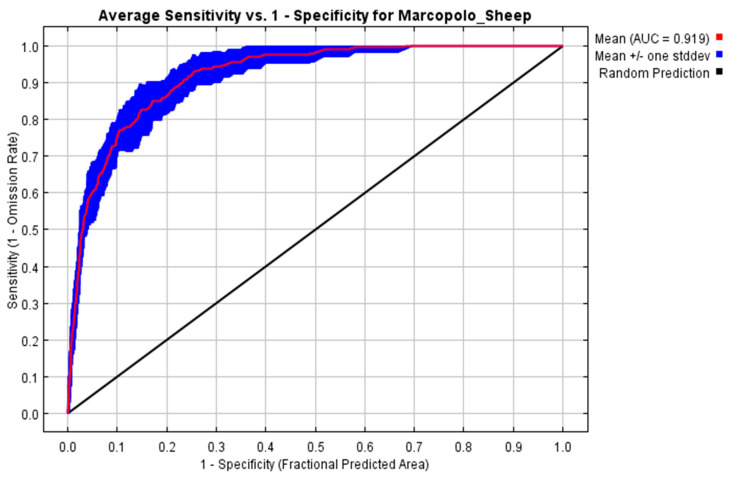
Assessing the predictive accuracy of ecological models for Marco Polo sheep through Receiver Operating Characteristic (ROC) analysis.

The ROC curve for the Marco Polo sheep habitat suitability model demonstrates high predictive performance, with an average AUC value of 0.919 and a standard deviation of 0.015 (Figure 2). These results indicate that the model is highly reliable and effective in identifying suitable habitats.

**Figure 3:**
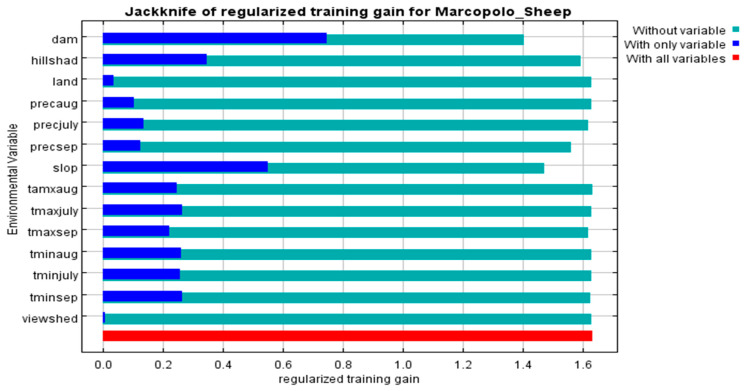

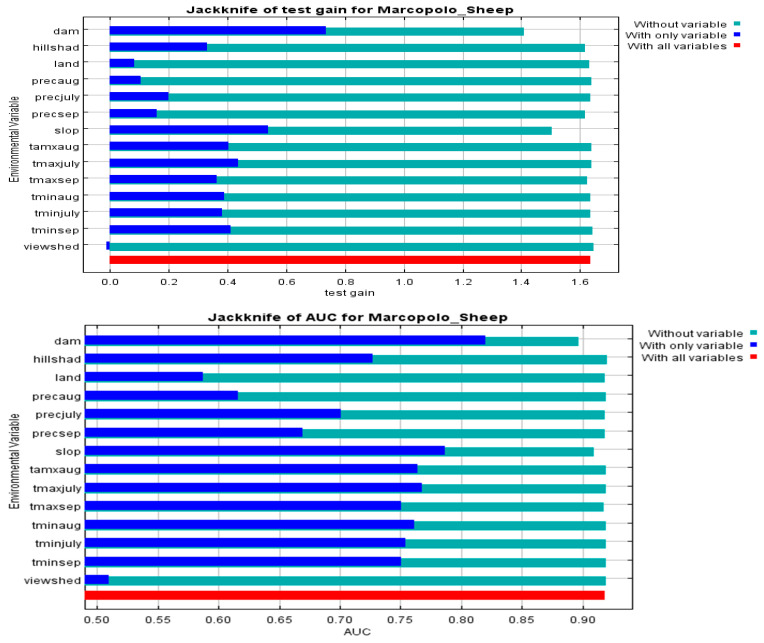
Jackknife test results of MaxEnt model of Marco Polo sheep (*Ovis ammon polii*) in Pakistan (KNP)–China (TNR) border region.

To evaluate the contribution of different environmental variables in the MaxEnt predictions, a Jackknife test was conducted (Figure 3). This test measured training and regularized training gains across three scenarios: using all variables, using one variable individually, and excluding one variable. Out of 14 environmental variables, elevation, slope, hillshade, and the maximum temperature in July emerged as the most significant factors influencing the distribution of Marco Polo sheep. Moderate contributions were observed for the maximum temperature in July and the maximum temperature in August. Conversely, variables such as viewshade, land cover, and precipitation in August contributed a minimal gain, suggesting that they had little impact on accurately predicting species distribution (Table 2, Figure 3).

### 3.2. Modeling the Potential Geographic Distribution of Marco Polo Sheep

**Figure 4:**
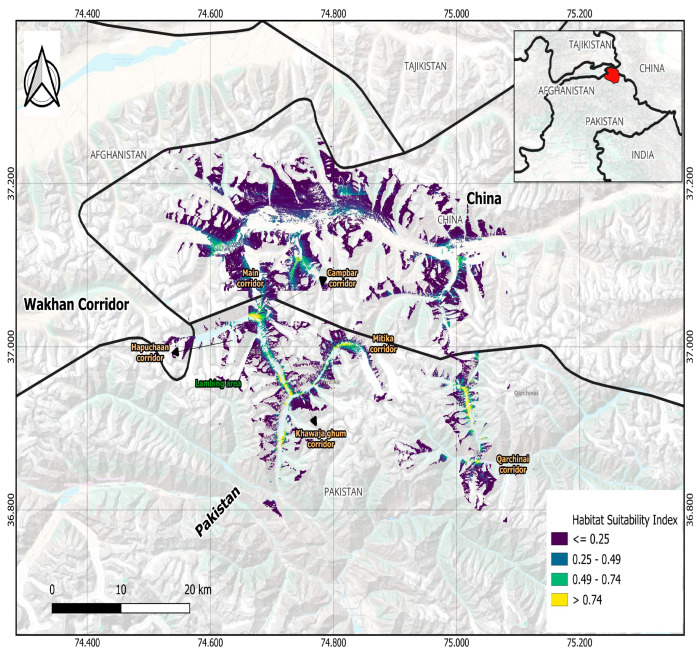
Habitat suitability and distribution map of Marco polo sheep (*Ovis ammon polii*) Pakistan (KNP)-China (TNR) border region.

The habitat suitability map employs a color gradient to depict varying levels of environmental suitability for Marco Polo sheep. Areas shaded in yellow denote the highest habitat suitability (≥0.75), indicating optimal ecological conditions. Green regions represent suitable habitats (0.49–0.74), while blue areas correspond to moderate suitability (0.25–0.49). Purple areas indicate low suitability (≤0.25), signifying suboptimal or unsuitable habitats (Figure 4). However, a region near the Wakhan Corridor—where the borders of Afghanistan, Pakistan, China, and Tajikistan converge—displays variable suitability, likely influenced by ecological and environmental factors.

### 3.3. Key Environmental Parameters Influencing Marco Polo Sheep Distribution

When modeling the geographic distribution of Marco Polo sheep, 14 environmental variables were assessed for their influence (Table 1). The top three contributors were elevation at 43.9%, slope at 25.9%, and annual precipitation at 15.9%, collectively accounting for approximately 85.7% of the model’s predictive capability. Permutation importance analysis further highlighted elevation as the most critical factor, with a score of 19.3, followed by September precipitation (precsep) at 16.9 and the maximum temperature in September (tmaxsep) at 16.5. In contrast, variables such as hillshade, land cover type, and viewshed had minimal impact, with permutation importance scores ranging from 0.1 to 2 (Table 2).

The environmental variables incorporated into the model included elevation, slope, and land cover, which provided insights into the topographical and vegetative characteristics of the study area. Precipitation metrics for August, July, and September were essential for understanding seasonal rainfall patterns. Temperature variables, specifically the maximum and minimum temperatures for August, July, and September, measured in degrees Celsius, offered a comprehensive view of the thermal environment. These variables were instrumental in accurately predicting habitat suitability for Marco Polo sheep (Table 1 and Table 2). It is important to note that while percent contribution values provide insights into variable importance, they are heuristically defined and can be influenced by correlations among variables. Therefore, interpretations should be made with caution, especially when variables are highly correlated.

### 3.4. Habitat Suitability Modeling and Threshold Optimization for Marco Polo Sheep Conservation

**Figure 5:**
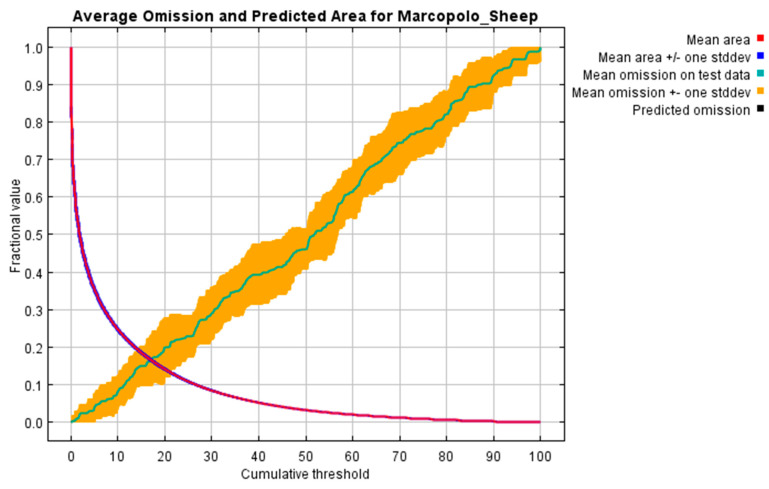
Mean and predicted omission rates for mean output of MaxEnt model of Marco Polo sheep.

The habitat suitability analysis for Marco Polo sheep reveals a trade-off between predicted area coverage and omission rates across increasing thresholds. Optimal model performance is achieved at intermediate thresholds (~30–50%), balancing habitat inclusion and prediction accuracy. Standard deviation bands indicate consistent model reliability. These findings provide a robust basis for conservation planning in the species’ range (Figure 5).

The detailed threshold analysis (Table 3) provided specific guidance for model implementation. The most inclusive threshold (1.0 cumulative value) predicted 59.9% of the study area with perfect omission rates (0% for both training and test data), while biologically informed thresholds like the 10-percentile training presence (17.528 cumulative value) offered a balanced compromise (16.3% area predicted, 9.5% test omission). Notably, all threshold criteria demonstrated statistically significant predictive performance (*p* < 1 × 10^−5^), with the “Balance” threshold (2.939 cumulative value) particularly effective at optimizing multiple criteria simultaneously (44.2% area predicted, 0.5% training omission).

## 4. Discussion

### 4.1. Environmental and Anthropogenic Influences on Marco Polo Sheep Distribution

Our study demonstrates that the distribution of Marco Polo sheep in the study area is significantly influenced by climatic variables such as elevation, slope, temperature, and precipitation [6,21,28,29]. These natural factors, such as climate change, along with anthropogenic threats including fencing, hunting, habitat fragmentation, and heightened predation risks during lambing season [1,7,16], are critical in shaping the behavior and ecological patterns of sheep in the study area. Together, these findings underscore the interplay between environmental variables and human interference in determining the sheep’s distribution and habitat suitability in their natural range [14].

Elevation and topographical features are the most significant drivers of habitat suitability for Marco Polo sheep. Snowmelt at high altitudes promotes the growth of protein-rich edible plants, attracting sheep to forage and lamb in these areas [9,21,35,36]. These high-altitude habitats, characterized by flat, undulating plateaus with smooth relief, also provide safety from predators, encouraging the sheep to cluster in large groups [37]. Slope (bio-7) influences habitat selection, with sheep preferring rolling hills and open areas with gentle or moderate slopes, as these terrains align with their thermoregulatory and foraging requirements [3,16,28]. This preference reflects the species’ ability to avoid extreme heat and predation by inhabiting suitable slopes, a strategy supported by the broader argali subspecies, which occupy elevations between 3000 and 5750 m [4,10], favoring open slopes interspersed with uneven patches [37] (Table 1 and Table 2).

Variables such as land cover and viewshed showed minimal influence on habitat selection (Table 2, Figure 3); however, human modifications to terrain, such as habitat fragmentation and fencing, are significant concerns [7,28]. Fencing restricts migration corridors, while changes in land cover lead to habitat fragmentation, negatively impacting the sheep population [7,9,14]. These pressures threaten the long-term survival of Marco Polo sheep, emphasizing the urgent need for conservation measures to address human-induced impacts [16].

### 4.2. Regional Habitat Suitability and Distribution

In the eastern region, extending towards Qarchanai and TNR, area of China, habitat suitability ranged from moderate to low [2,10,11], with the suitability map showing a mix of purple and blue, interspersed with patches of green and small yellow areas along the central eastern ridges. The west Wakhan Corridor, located along the Afghanistan border, offers high to very high habitat suitability [2,3,38], marked by dense green, blue, and continuous yellow belts along the ridges. In the northern region bordering China [3] and Tajikistan, habitat suitability was found to be generally high to moderate [2,5,28], with the presence of green, blue, and notable yellow patches, particularly along the central northern ridgelines [1,17]. In the southern region, encompassing Khunjerab Pass and the Misgar area of Pakistan, habitat suitability was predominantly low [2,4,10,14], as indicated by the dominance of purple shades on the suitability map, with only sparse patches of green and blue (Figure 4. The best habitats, marked by yellow zones, are located in the western Wakhan Corridor and northern central ridgelines. Poor habitats, represented by purple zones, are concentrated in the southern regions, particularly near Khunjerab Pass (Figure 4).

### 4.3. Habitat Suitability Across the Study Area Varies Significantly

Eastern Regions: These regions exhibit moderate to low habitat suitability, offering moderate food resources, shelter, and minimal human disturbance [23]; however areas characterized by steep slopes and rugged mountainous terrain [14] provide critical refuge during lambing seasons by enabling escape from predators [4,10]. Qarchanai, historically a critical habitat, has seen a decline in population, with sightings dropping from 52 individuals in 1989 to 38 in 2011 [2,17]. Seasonal migrations for lambing occur in these areas, with females and subadult males moving to Qarchanai in late May and returning to Taxkorgan, China, in mid-September [1,4] (Figure 4).

Western Regions: The west Wakhan Corridor shows high habitat suitability due to its dense vegetation and water resources, but this potential is undermined by heavy habitat fragmentation from anthropogonic disturbances and human activities like livestock grazing [9]. While the ridges form continuous ecological belts, valley bottoms and slopes face degradation from overgrazing and fuelwood collection [10,14]. For example, Chalachigu Valley supports 284 individuals, while Pisilang recorded the highest population of 436 individuals [1,39]. This creates a mosaic of suitable patches isolated by degraded zones, limiting wildlife movement and long-term viability. Effective conservation here requires addressing both ecological connectivity and anthropogenic pressures to maintain its role as a biodiversity hotspot [2] (Figure 4).

Northern and Southern Regions: Northern areas like Mustagh Ata and Kongur Range exhibit high to moderate suitability but are constrained by fencing and limited forage in sub-catchments [14]. Southern regions are characterized by poor to moderate suitability due to barren terrain, water scarcity, and poaching. While high densities have been recorded in Pisilang, these findings contradict our results, indicating the need for further investigation [2] (Figure 4).

Overall, the best habitats, marked by yellow zones, are located in the western Wakhan Corridor and northern central ridgelines. Poor habitats, represented by purple zones, are concentrated in the southern regions, particularly near Khunjerab Pass (Figure 4). Conservation priority should focus on the yellow and green zones across the north and west, emphasizing protection, species monitoring, and connectivity to ensure long-term sustainability [4,7,9,32] (Figure 4).

The Jackknife test (Figure 3) highlights the significant contributions of climatic variables such as bio-1 (elevation), bio-2 (hillshade), bio-7 (slope), and bio-9 (maximum temperature) to habitat suitability [19,29,40,41]. Seasonal precipitation (bio-4, bio-5, bio-6) plays a critical role in shaping species distribution and abundance, emphasizing the importance of rainfall patterns in ecological studies [18,42]. Temperature variables (bio-8 to bio-13) further elucidate the species’ thermal preferences and tolerances, underscoring the role of temperature regimes in influencing habitat suitability [29,43]. In contrast, variables like viewshed, landcover, and precipitation in August exhibited lower predictive power, indicating that these factors are less critical for habitat selection in high-elevation species [43,44].

These results indicate that Marco Polo sheep select both the abiotic and biotic features of their environment, including parklands, areas with high solar exposure, rugged terrain, and landscapes that facilitate escape from predators [45]. The habitat choices of Marco Polo sheep during the spring period are based on progressions in vegetation and temperature, but their winter choices primarily center around snow protection and extreme cold temperatures [46]. Overall, Marco Polo sheep demonstrate flexible responses to both environmental and anthropogenic pressures. Variations across populations reveal that a range of environmental factors influence their habitat selection strategies [47].

The integration of these variables in geographic distribution models enhances our understanding of Marco Polo sheep’s ecological niche and habitat requirements [21,34,48]. The strong performance of our habitat suitability model, with an AUC value of 0.919 (Figure 2), demonstrates its effectiveness in predicting suitable habitats for the species in challenging mountainous terrains [22]. In comparison to previous studies of Phillips et al. [48] and Evcin et al. [20], our model shows even stronger predictive power, reflecting the comprehensive inclusion of critical environmental variables (Figure 2).

### 4.4. Habitat Suitability Thresholds and Conservation Implications

The minimum training presence method predicted 59.9% of the study area as a suitable habitat for Marco Polo sheep (Table 3). However, ongoing fencing, road construction [4,8,10,16], and overgrazing within buffer zones threaten to reduce this habitat [15,33], potentially confining the species to small, isolated patches that may no longer meet their breeding and foraging needs. For instance, livestock grazing in areas like Kilik and Mintika exacerbates resource depletion, converting grazing areas into degraded landscapes [15,33].

Our findings align with previous studies [1,14,20], emphasizing the urgency of conservation measures to protect and restore suitable habitats. Efforts should focus on mitigating the impacts of fencing, human encroachment, and resource competition while maintaining connectivity between critical habitats. These steps will ensure the long-term survival of Marco Polo sheep in their transboundary range between China (TNR) and Pakistan (KNP) [7,9]. Additional details and supporting data are provided in the Supplementary Materials available at https://www.mdpi.com/article/10.3390/ani15131907/s1.

## 5. Conclusions

This study identifies elevation, slope, and seasonal precipitation as the most critical drivers of habitat suitability for the endangered Marco Polo sheep (*Ovis ammon polii*), with fencing and anthropogenic pressures exacerbating habitat fragmentation in key regions. Our high-accuracy MaxEnt model (AUC = 0.919) reveals optimal habitats in the Wakhan Corridor and northern ridgelines, while southern areas face severe degradation due to human activities. The findings underscore an urgent need for transboundary conservation strategies between Pakistan and China, prioritizing habitat connectivity, anti-poaching measures, and climate-resilient management. By integrating these science-based interventions, stakeholders can mitigate escalating threats and safeguard this iconic species in its high-mountain ecosystem.

To further fortify long-term conservation efficacy, a broader suite of protection management strategies should be considered. These may include the formal designation of ecological corridors, the institutionalization of community-based conservation governance, the regulation of livestock densities in sensitive zones, and the development of sustainable eco-tourism models that incentivize preservation through local economic integration.

## Figures and Tables

**Table 1:** Environmental variables were used to predict geographic distribution models for the Marco Polo sheep (*Ovis ammon polii*) in the Pakistan (KNP)–China (TNR) border region.

S/NO	Description of the Variables	Unit	Resolution	Source
1	Elevation/DEM	M	30 m	USGS Earth data portal
2	Hillshed	M	30 m	By calculation
3	Land cover	Km^2^	10 m	ESRI
4	Precipitation (Aug)	Mm	2.5 min	WorldClim
5	Precipitation (July)	Mm	2.5 min	WorldClim
6	Precipitation (Sep)	Mm	2.5 min	WorldClim
7	Slop	“°”	30 m	By calculation
8	Maximum temperature (Aug)	°C	2.5 min	WorldClim
9	Maximum temperature (July)	°C	2.5 min	WorldClim
10	Minimum temperature (Sep)	°C	2.5 min	WorldClim
11	Minimum temperature (Aug)	°C	2.5 min	WorldClim
12	Minimum temperature (July)	°C	2.5 min	WorldClim
13	Maximum temperature (Sep)	°C	2.5 min	WorldClim
14	Viewshed	M	30 m	By calculation

Note: The climate variables used in the model.

**Table 2:** The percent contribution of environmental variables in predicting geographic distribution models for Marco Polo sheep.

Variable	Percent Contribution	Permutation Importance
elevation	43.9	19.3
slop	25.9	11.8
precsep	15.9	16.9
hillshed	4.7	2
tamxaug	3.2	6.5
tminsep	1.9	3.6
tminjuly	1.4	0.6
tmaxsep	0.9	16.5
tminaug	0.8	0.6
tmaxjuly	0.7	15.9
precjuly	0.3	5.7
land cover	0.2	0.1
viewshed	0.1	0.1
precaug	0	0.4

Note: Environmental variables are arranged in order of contributions.

**Table 3:** Some common thresholds and corresponding omission rates of Marco Polo sheep (*Ovis ammon polii*) in Pakistan (KNP)–China (TNR) border region.

Cumulative Threshold	Cloglog Threshold	Description	Fractional Predicted Area	Training Omission Rate	Test Omission Rate	*p*-Value
1.000	0.015	Fixed cumulative value 1	0.599	0.000	0.000	2.105 × 10^−5^
5.000	0.060	Fixed cumulative value 5	0.360	0.016	0.000	4.768 × 10^−10^
10.000	0.117	Fixed cumulative value 10	0.250	0.054	0.095	4.467 × 10^−10^
1.921	0.025	Minimum training presence	0.506	0.000	0.000	6.136 × 10^−7^
17.528	0.199	10-percentile training presence	0.163	0.097	0.095	1.629 × 10^−13^
21.013	0.239	Equal training sensitivity and specificity	0.135	0.135	0.143	1.983 × 10^−13^
17.233	0.194	Maximum training sensitivity plus specificity	0.166	0.081	0.095	2.199 × 10^−13^
19.975	0.225	Equal test sensitivity and specificity	0.143	0.135	0.143	5.281 × 10^−13^
19.899	0.224	Maximum test sensitivity plus specificity	0.143	0.130	0.095	1.467 × 10^−14^
2.939	0.037	Balance training omission, predicted area, and threshold value	0.442	0.005	0.000	3.617 × 10^−8^
14.161	0.163	Equate entropy of thresholded and original distributions	0.196	0.081	0.095	5.191 × 10^−12^

## Data Availability

Data will be made available on request.

## Supplementary Materials

The following supporting information can be downloaded at: https://www.mdpi.com/article/10.3390/ani15131907/s1.
