# Supplementary figures and images for: Assessment of Habitat Suitability and Identification of Conservation Priority Areas for Endangered Marco Polo Sheep Throughout Khunjerab National Park (Pakistan) and Tashkurgan Natural Reserve (China) (part 2 of 2)
Source: Animals (Basel). 2025 Jun 28;15(13):1907. doi: 10.3390/ani15131907 (PMC12248910; doi:10.3390/ani15131907)

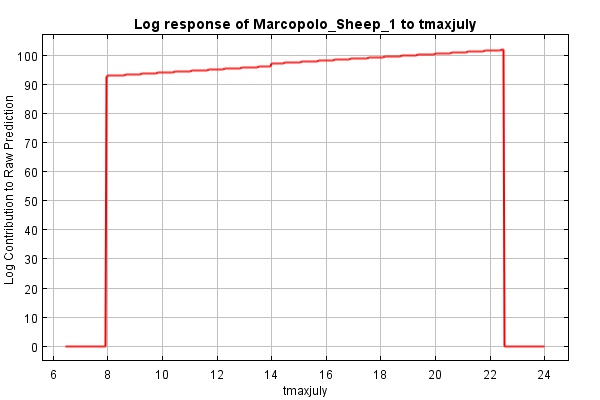

Supplement: Supplementary file 1 [file animals-15-01907-s001.zip › plots/Marcopolo_Sheep_1_tmaxjuly.png]

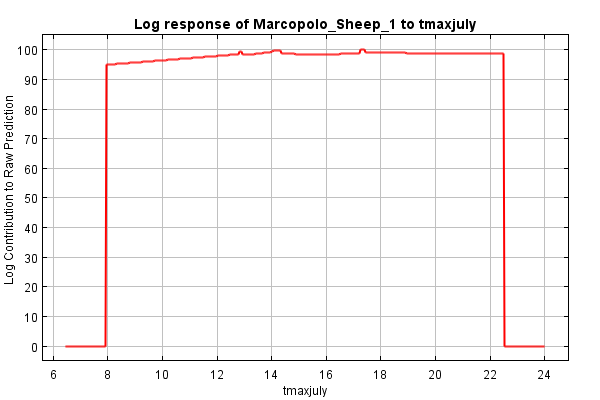

Supplement: Supplementary file 1 [file animals-15-01907-s001.zip › plots/Marcopolo_Sheep_1_tmaxjuly_only.png]

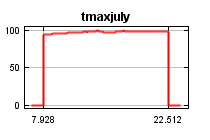

Supplement: Supplementary file 1 [file animals-15-01907-s001.zip › plots/Marcopolo_Sheep_1_tmaxjuly_only_thumb.png]

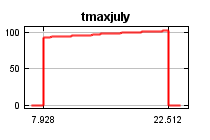

Supplement: Supplementary file 1 [file animals-15-01907-s001.zip › plots/Marcopolo_Sheep_1_tmaxjuly_thumb.png]

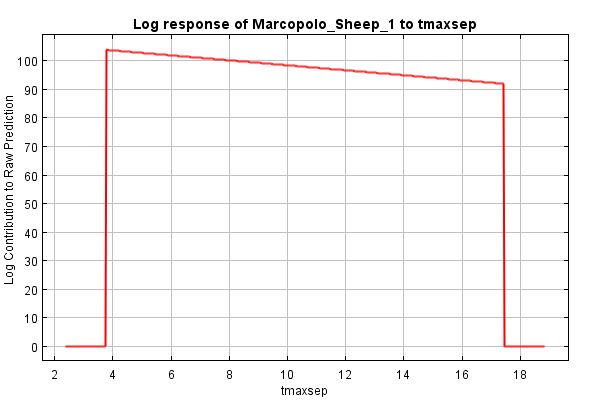

Supplement: Supplementary file 1 [file animals-15-01907-s001.zip › plots/Marcopolo_Sheep_1_tmaxsep.png]

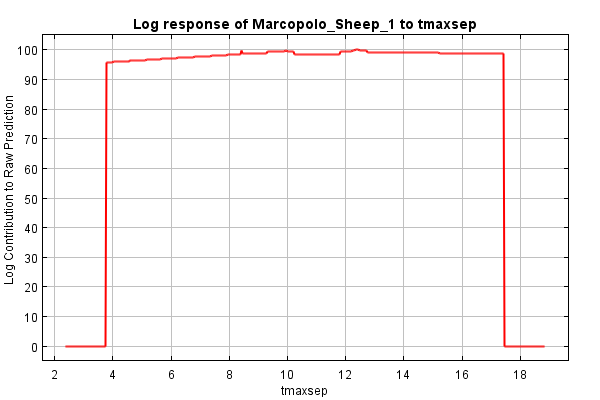

Supplement: Supplementary file 1 [file animals-15-01907-s001.zip › plots/Marcopolo_Sheep_1_tmaxsep_only.png]

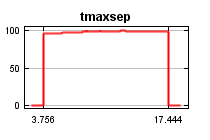

Supplement: Supplementary file 1 [file animals-15-01907-s001.zip › plots/Marcopolo_Sheep_1_tmaxsep_only_thumb.png]

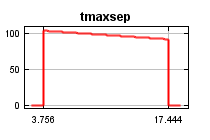

Supplement: Supplementary file 1 [file animals-15-01907-s001.zip › plots/Marcopolo_Sheep_1_tmaxsep_thumb.png]

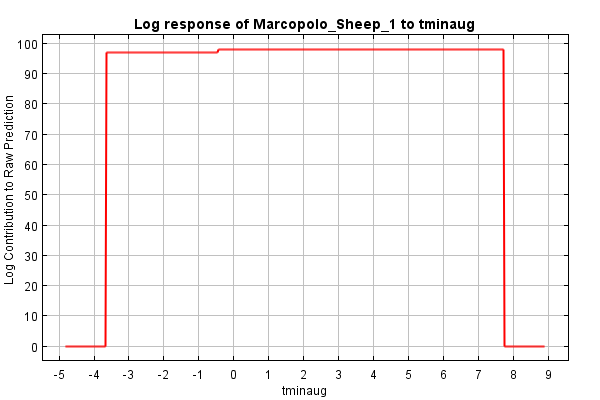

Supplement: Supplementary file 1 [file animals-15-01907-s001.zip › plots/Marcopolo_Sheep_1_tminaug.png]

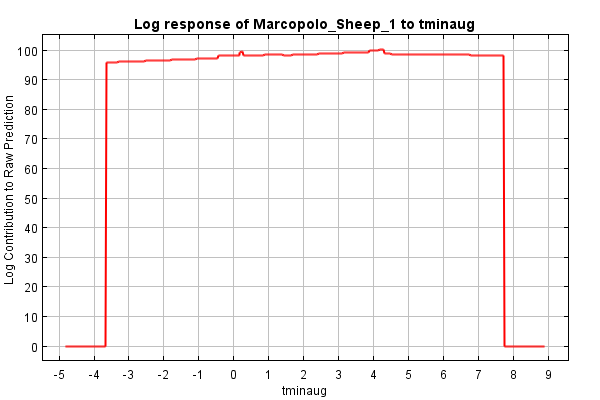

Supplement: Supplementary file 1 [file animals-15-01907-s001.zip › plots/Marcopolo_Sheep_1_tminaug_only.png]

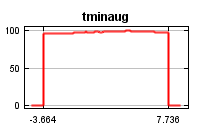

Supplement: Supplementary file 1 [file animals-15-01907-s001.zip › plots/Marcopolo_Sheep_1_tminaug_only_thumb.png]

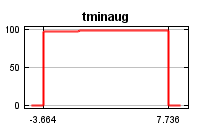

Supplement: Supplementary file 1 [file animals-15-01907-s001.zip › plots/Marcopolo_Sheep_1_tminaug_thumb.png]

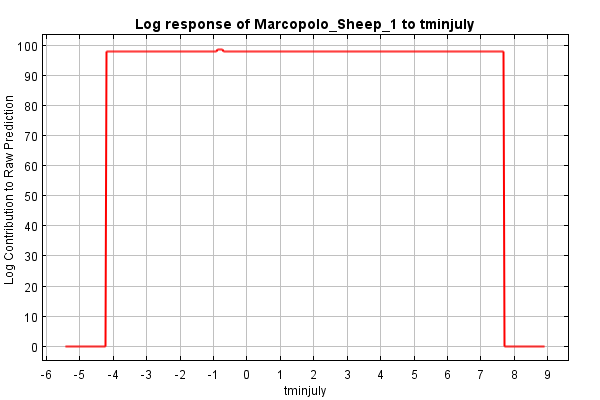

Supplement: Supplementary file 1 [file animals-15-01907-s001.zip › plots/Marcopolo_Sheep_1_tminjuly.png]

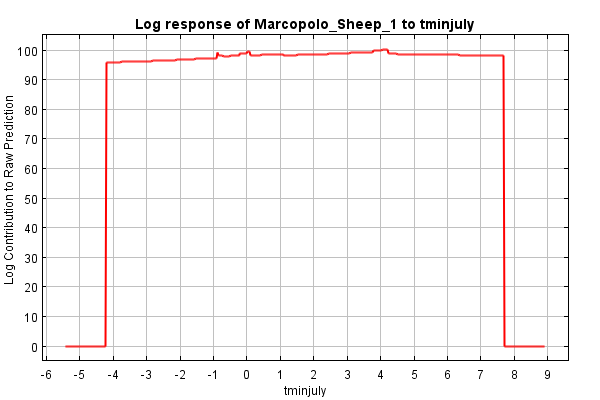

Supplement: Supplementary file 1 [file animals-15-01907-s001.zip › plots/Marcopolo_Sheep_1_tminjuly_only.png]

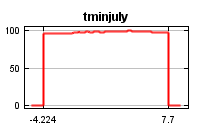

Supplement: Supplementary file 1 [file animals-15-01907-s001.zip › plots/Marcopolo_Sheep_1_tminjuly_only_thumb.png]

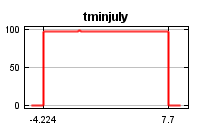

Supplement: Supplementary file 1 [file animals-15-01907-s001.zip › plots/Marcopolo_Sheep_1_tminjuly_thumb.png]

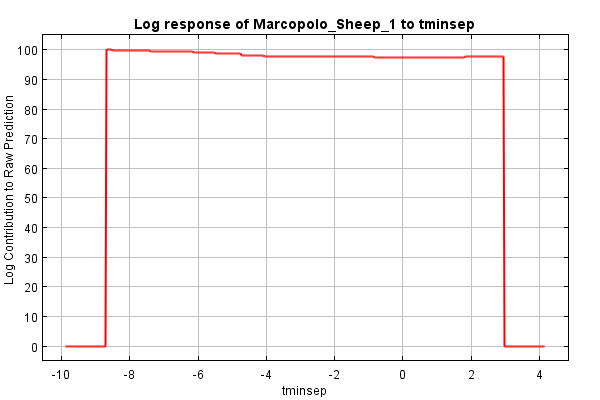

Supplement: Supplementary file 1 [file animals-15-01907-s001.zip › plots/Marcopolo_Sheep_1_tminsep.png]

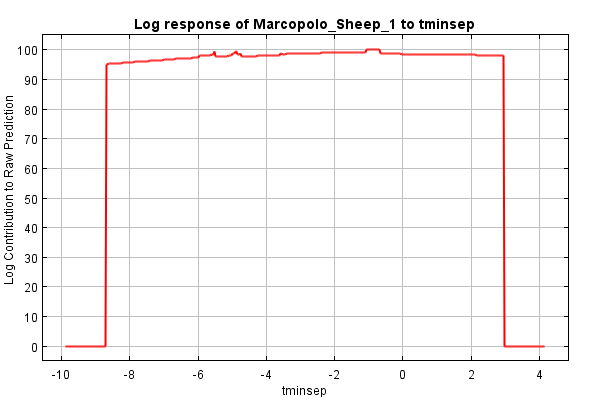

Supplement: Supplementary file 1 [file animals-15-01907-s001.zip › plots/Marcopolo_Sheep_1_tminsep_only.png]

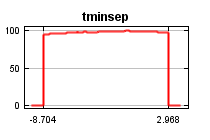

Supplement: Supplementary file 1 [file animals-15-01907-s001.zip › plots/Marcopolo_Sheep_1_tminsep_only_thumb.png]

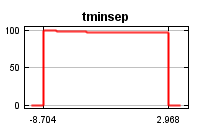

Supplement: Supplementary file 1 [file animals-15-01907-s001.zip › plots/Marcopolo_Sheep_1_tminsep_thumb.png]

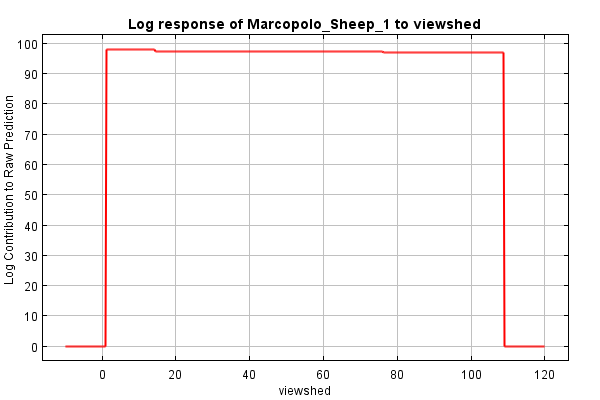

Supplement: Supplementary file 1 [file animals-15-01907-s001.zip › plots/Marcopolo_Sheep_1_viewshed.png]

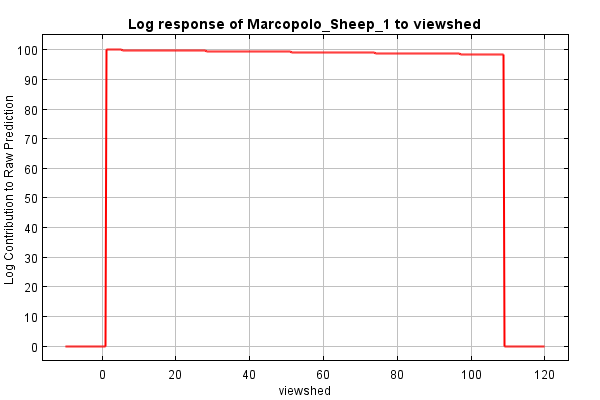

Supplement: Supplementary file 1 [file animals-15-01907-s001.zip › plots/Marcopolo_Sheep_1_viewshed_only.png]

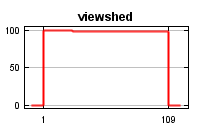

Supplement: Supplementary file 1 [file animals-15-01907-s001.zip › plots/Marcopolo_Sheep_1_viewshed_only_thumb.png]

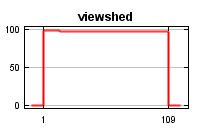

Supplement: Supplementary file 1 [file animals-15-01907-s001.zip › plots/Marcopolo_Sheep_1_viewshed_thumb.png]

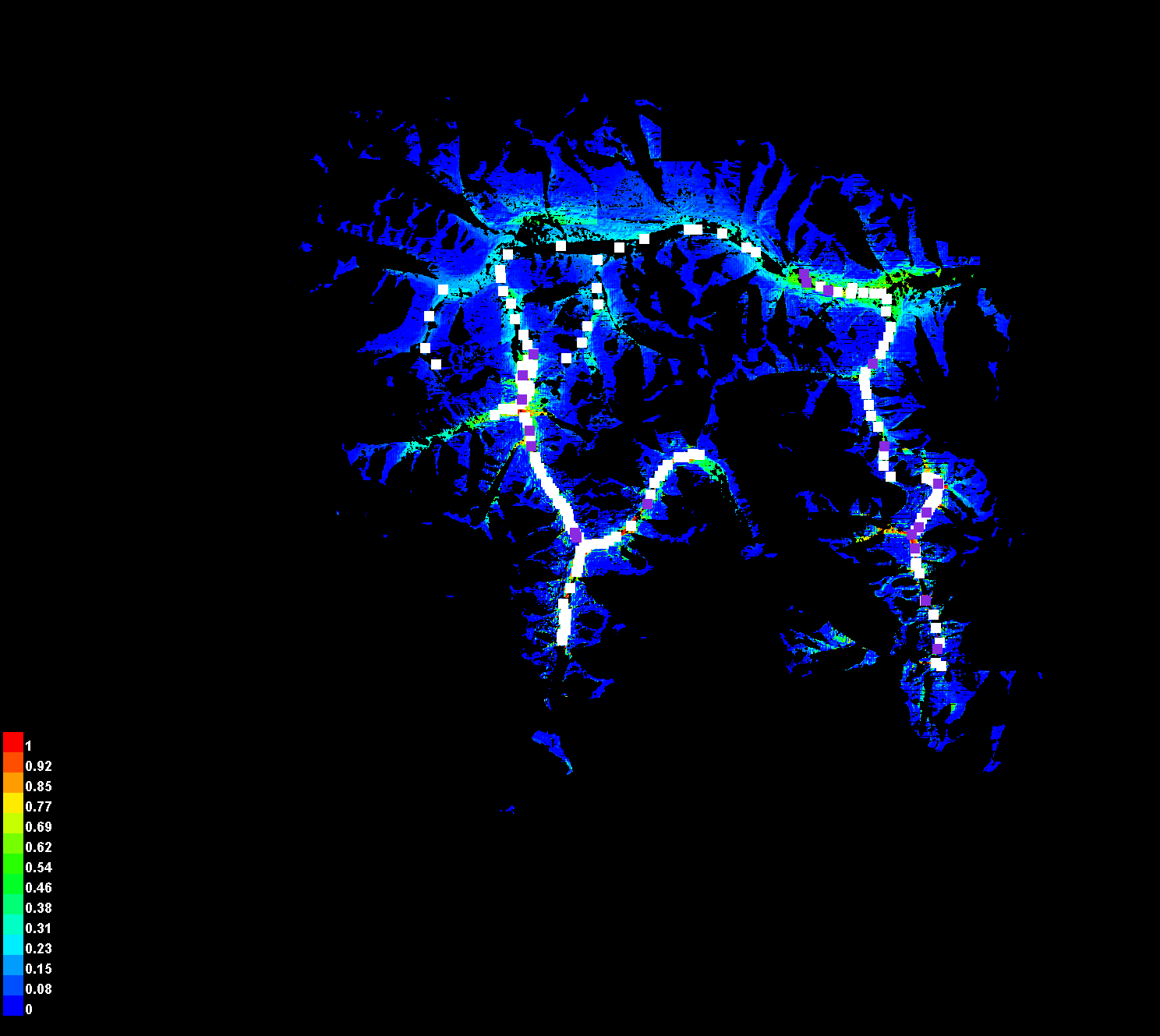

Supplement: Supplementary file 1 [file animals-15-01907-s001.zip › plots/Marcopolo_Sheep_2.png]

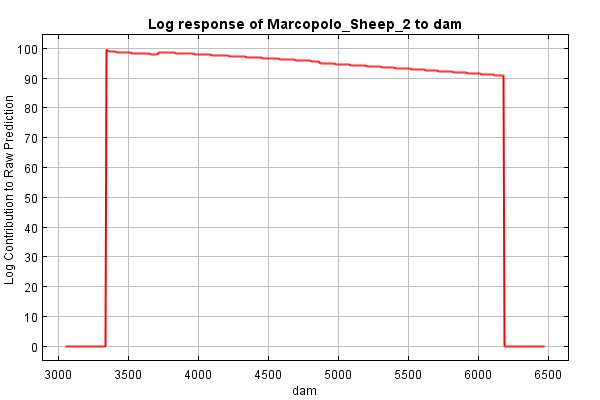

Supplement: Supplementary file 1 [file animals-15-01907-s001.zip › plots/Marcopolo_Sheep_2_dam.png]

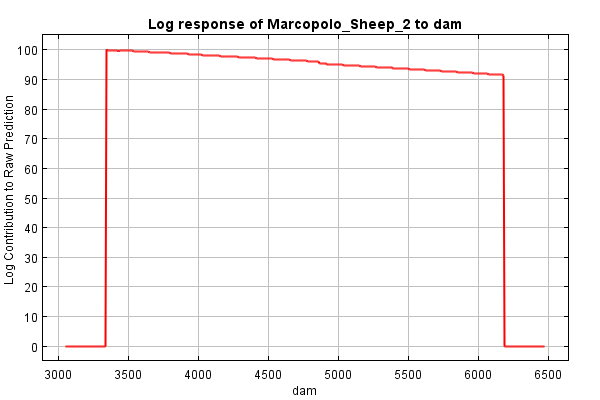

Supplement: Supplementary file 1 [file animals-15-01907-s001.zip › plots/Marcopolo_Sheep_2_dam_only.png]

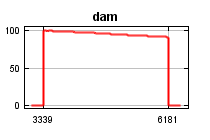

Supplement: Supplementary file 1 [file animals-15-01907-s001.zip › plots/Marcopolo_Sheep_2_dam_only_thumb.png]

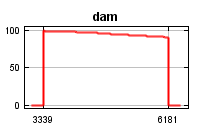

Supplement: Supplementary file 1 [file animals-15-01907-s001.zip › plots/Marcopolo_Sheep_2_dam_thumb.png]

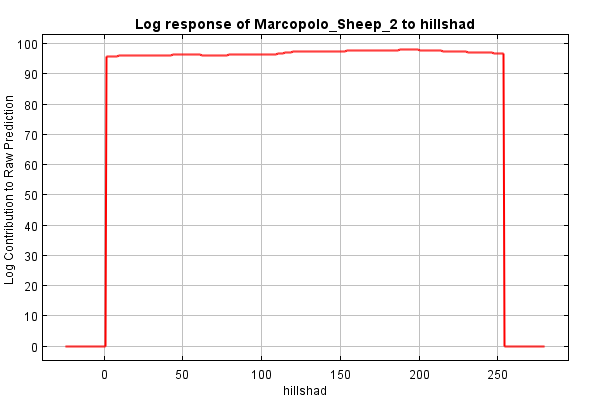

Supplement: Supplementary file 1 [file animals-15-01907-s001.zip › plots/Marcopolo_Sheep_2_hillshad.png]

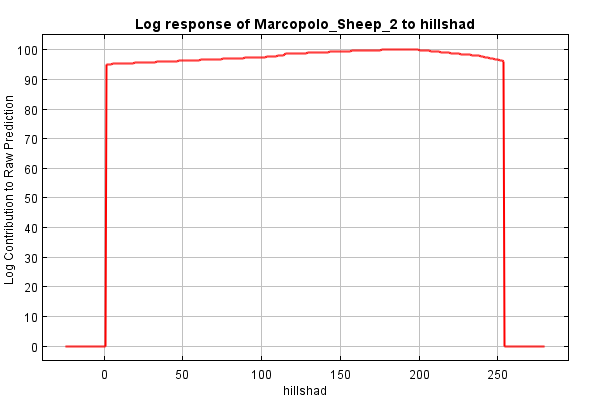

Supplement: Supplementary file 1 [file animals-15-01907-s001.zip › plots/Marcopolo_Sheep_2_hillshad_only.png]

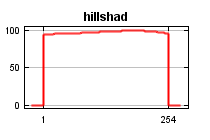

Supplement: Supplementary file 1 [file animals-15-01907-s001.zip › plots/Marcopolo_Sheep_2_hillshad_only_thumb.png]

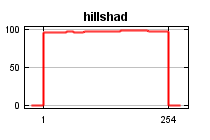

Supplement: Supplementary file 1 [file animals-15-01907-s001.zip › plots/Marcopolo_Sheep_2_hillshad_thumb.png]

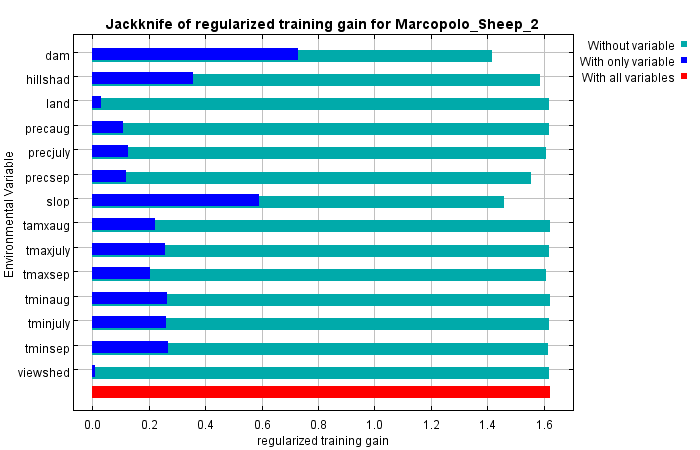

Supplement: Supplementary file 1 [file animals-15-01907-s001.zip › plots/Marcopolo_Sheep_2_jacknife.png]

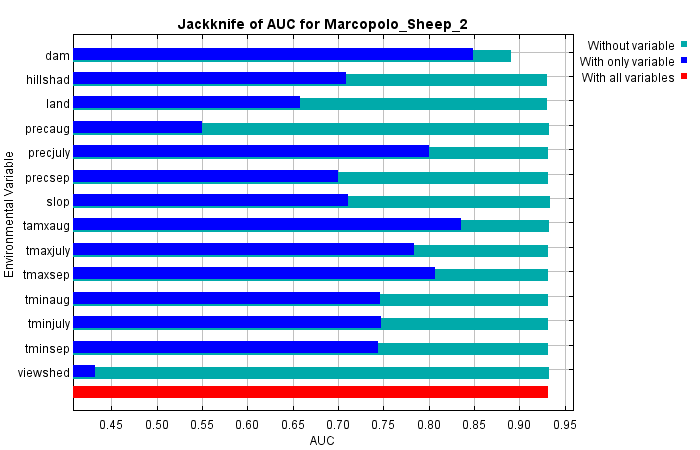

Supplement: Supplementary file 1 [file animals-15-01907-s001.zip › plots/Marcopolo_Sheep_2_jacknife_auc.png]

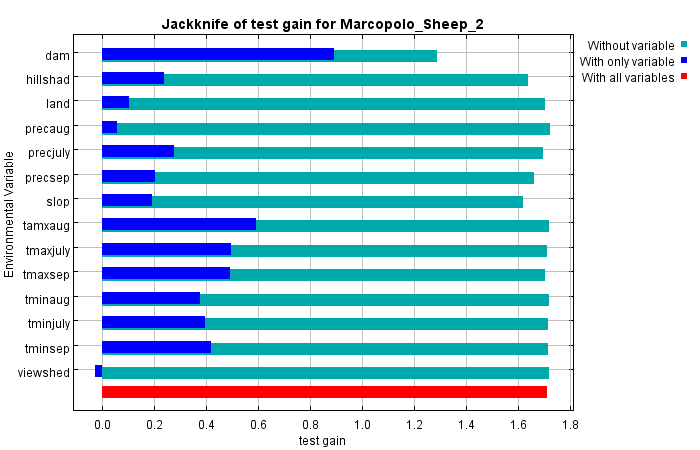

Supplement: Supplementary file 1 [file animals-15-01907-s001.zip › plots/Marcopolo_Sheep_2_jacknife_test.png]

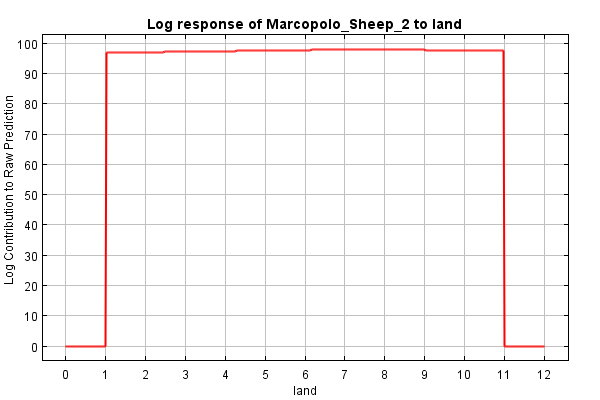

Supplement: Supplementary file 1 [file animals-15-01907-s001.zip › plots/Marcopolo_Sheep_2_land.png]
