# Supplementary figures and images for: Assessment of Habitat Suitability and Identification of Conservation Priority Areas for Endangered Marco Polo Sheep Throughout Khunjerab National Park (Pakistan) and Tashkurgan Natural Reserve (China) (part 1 of 2)
Source: Animals (Basel). 2025 Jun 28;15(13):1907. doi: 10.3390/ani15131907 (PMC12248910; doi:10.3390/ani15131907)

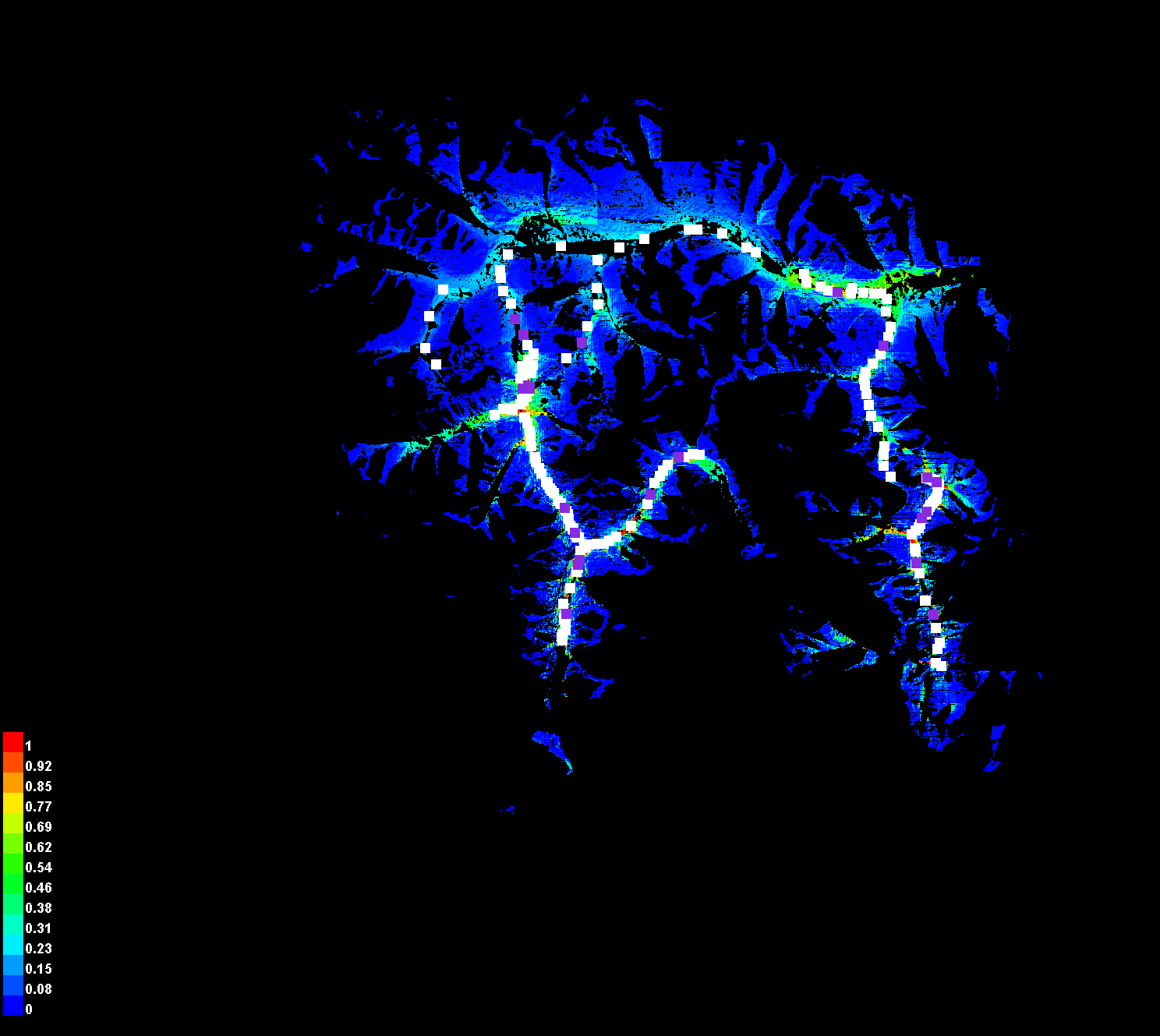

Supplement: Supplementary file 1 [file animals-15-01907-s001.zip › plots/Marcopolo_Sheep_0.png]

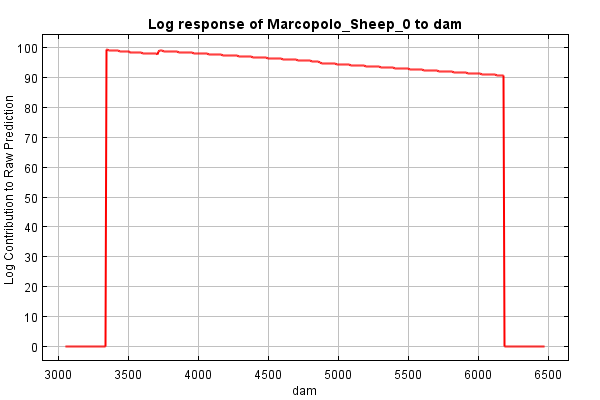

Supplement: Supplementary file 1 [file animals-15-01907-s001.zip › plots/Marcopolo_Sheep_0_dam.png]

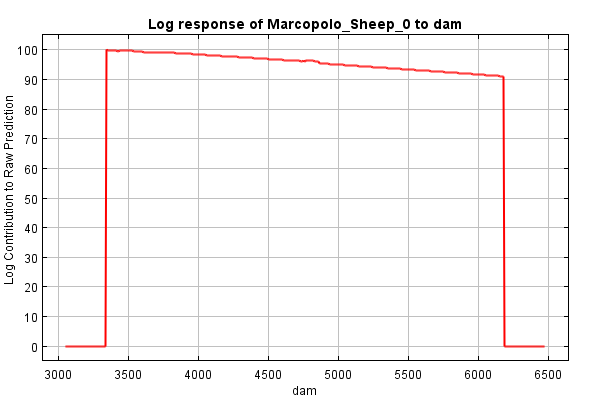

Supplement: Supplementary file 1 [file animals-15-01907-s001.zip › plots/Marcopolo_Sheep_0_dam_only.png]

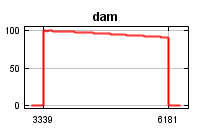

Supplement: Supplementary file 1 [file animals-15-01907-s001.zip › plots/Marcopolo_Sheep_0_dam_only_thumb.png]

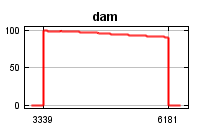

Supplement: Supplementary file 1 [file animals-15-01907-s001.zip › plots/Marcopolo_Sheep_0_dam_thumb.png]

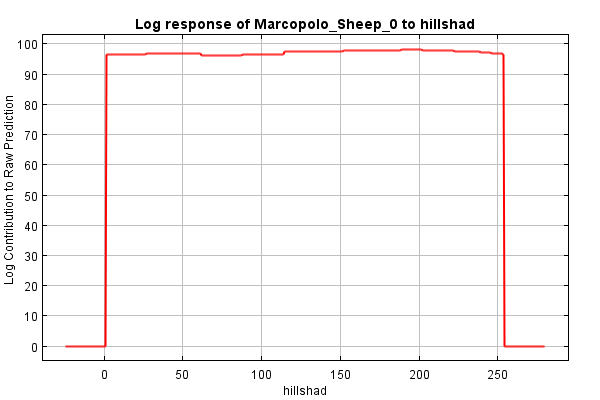

Supplement: Supplementary file 1 [file animals-15-01907-s001.zip › plots/Marcopolo_Sheep_0_hillshad.png]

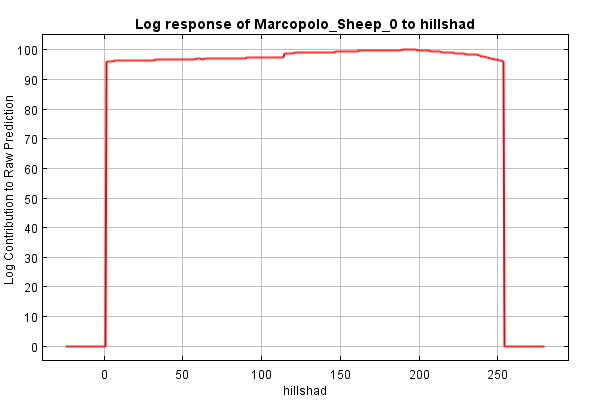

Supplement: Supplementary file 1 [file animals-15-01907-s001.zip › plots/Marcopolo_Sheep_0_hillshad_only.png]

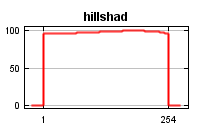

Supplement: Supplementary file 1 [file animals-15-01907-s001.zip › plots/Marcopolo_Sheep_0_hillshad_only_thumb.png]

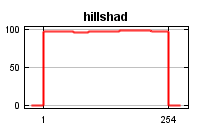

Supplement: Supplementary file 1 [file animals-15-01907-s001.zip › plots/Marcopolo_Sheep_0_hillshad_thumb.png]

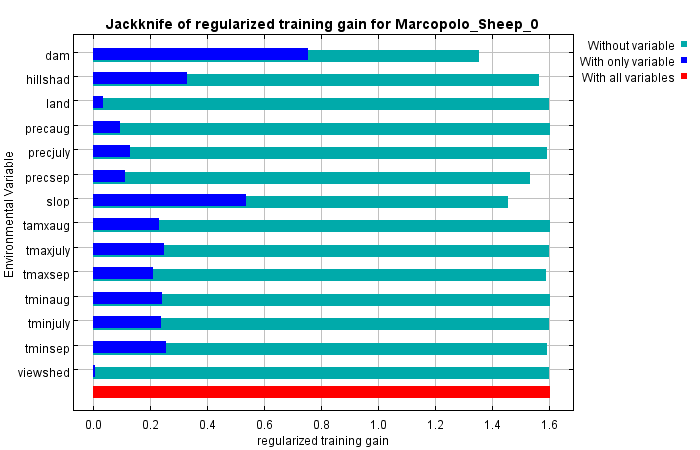

Supplement: Supplementary file 1 [file animals-15-01907-s001.zip › plots/Marcopolo_Sheep_0_jacknife.png]

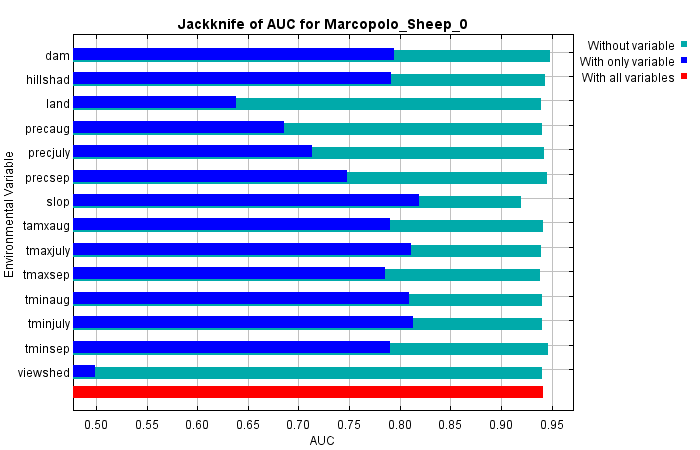

Supplement: Supplementary file 1 [file animals-15-01907-s001.zip › plots/Marcopolo_Sheep_0_jacknife_auc.png]

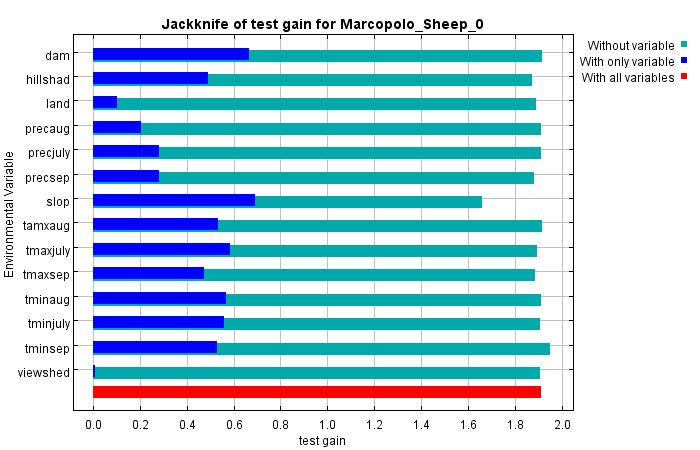

Supplement: Supplementary file 1 [file animals-15-01907-s001.zip › plots/Marcopolo_Sheep_0_jacknife_test.png]

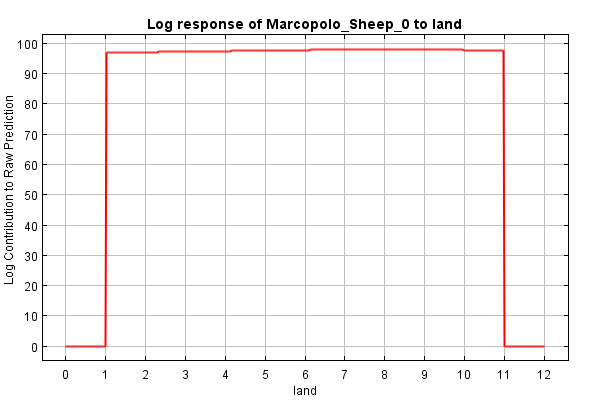

Supplement: Supplementary file 1 [file animals-15-01907-s001.zip › plots/Marcopolo_Sheep_0_land.png]

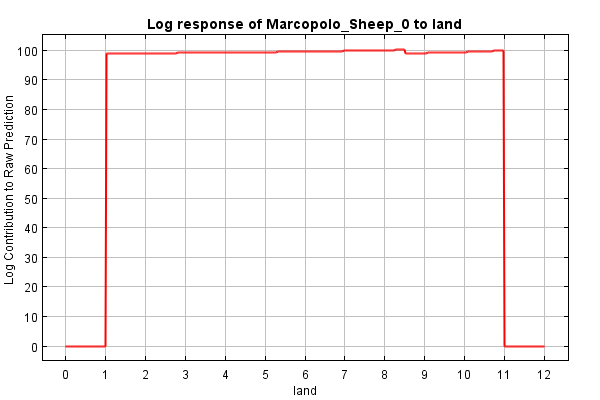

Supplement: Supplementary file 1 [file animals-15-01907-s001.zip › plots/Marcopolo_Sheep_0_land_only.png]

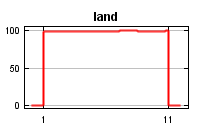

Supplement: Supplementary file 1 [file animals-15-01907-s001.zip › plots/Marcopolo_Sheep_0_land_only_thumb.png]

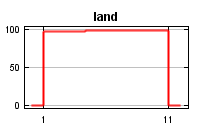

Supplement: Supplementary file 1 [file animals-15-01907-s001.zip › plots/Marcopolo_Sheep_0_land_thumb.png]

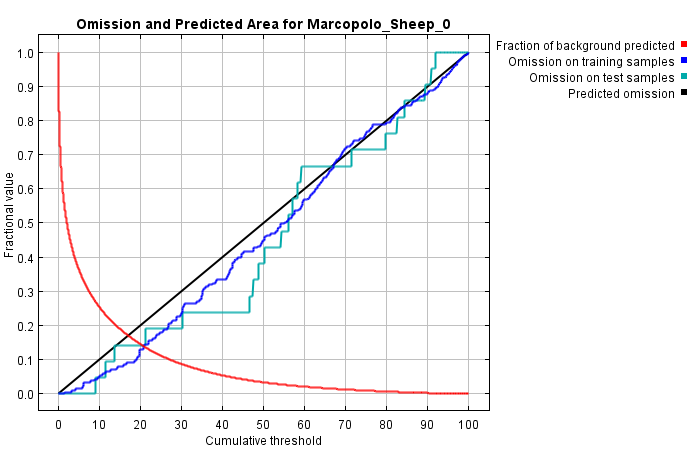

Supplement: Supplementary file 1 [file animals-15-01907-s001.zip › plots/Marcopolo_Sheep_0_omission.png]

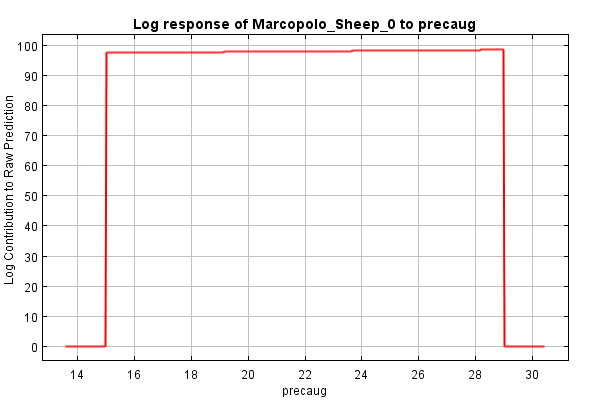

Supplement: Supplementary file 1 [file animals-15-01907-s001.zip › plots/Marcopolo_Sheep_0_precaug.png]

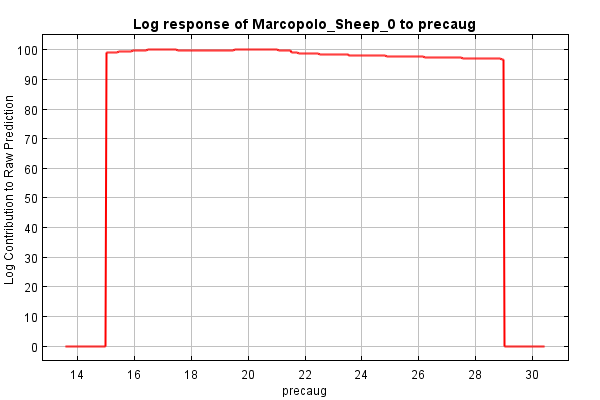

Supplement: Supplementary file 1 [file animals-15-01907-s001.zip › plots/Marcopolo_Sheep_0_precaug_only.png]

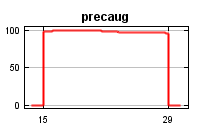

Supplement: Supplementary file 1 [file animals-15-01907-s001.zip › plots/Marcopolo_Sheep_0_precaug_only_thumb.png]

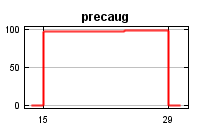

Supplement: Supplementary file 1 [file animals-15-01907-s001.zip › plots/Marcopolo_Sheep_0_precaug_thumb.png]

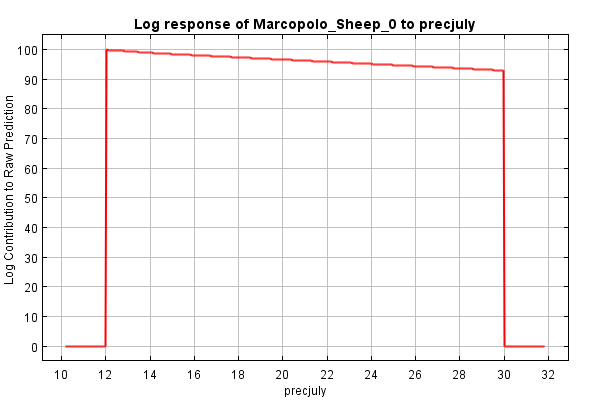

Supplement: Supplementary file 1 [file animals-15-01907-s001.zip › plots/Marcopolo_Sheep_0_precjuly.png]

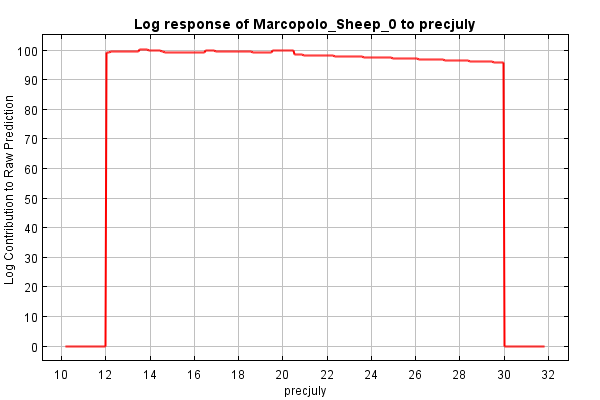

Supplement: Supplementary file 1 [file animals-15-01907-s001.zip › plots/Marcopolo_Sheep_0_precjuly_only.png]

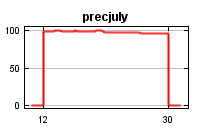

Supplement: Supplementary file 1 [file animals-15-01907-s001.zip › plots/Marcopolo_Sheep_0_precjuly_only_thumb.png]

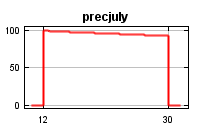

Supplement: Supplementary file 1 [file animals-15-01907-s001.zip › plots/Marcopolo_Sheep_0_precjuly_thumb.png]

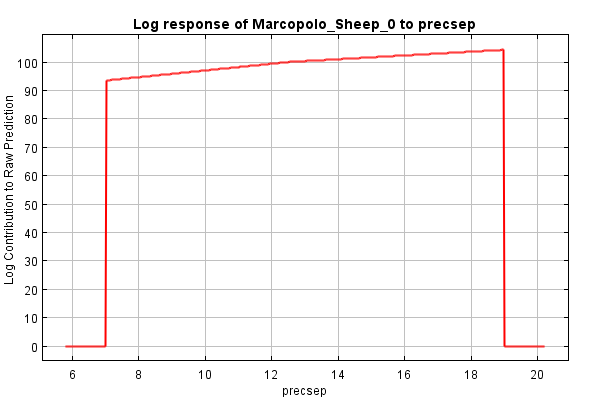

Supplement: Supplementary file 1 [file animals-15-01907-s001.zip › plots/Marcopolo_Sheep_0_precsep.png]

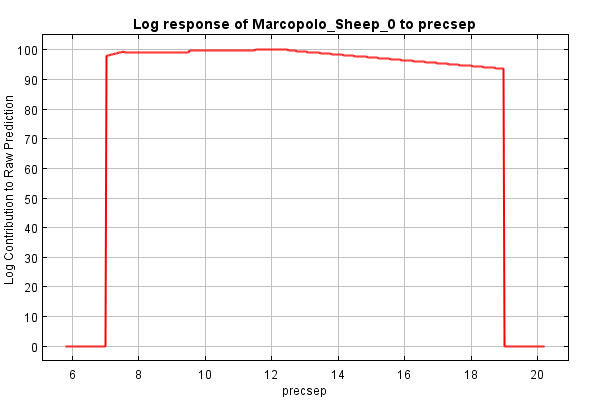

Supplement: Supplementary file 1 [file animals-15-01907-s001.zip › plots/Marcopolo_Sheep_0_precsep_only.png]

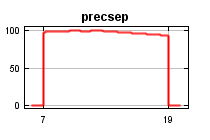

Supplement: Supplementary file 1 [file animals-15-01907-s001.zip › plots/Marcopolo_Sheep_0_precsep_only_thumb.png]

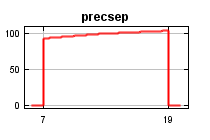

Supplement: Supplementary file 1 [file animals-15-01907-s001.zip › plots/Marcopolo_Sheep_0_precsep_thumb.png]

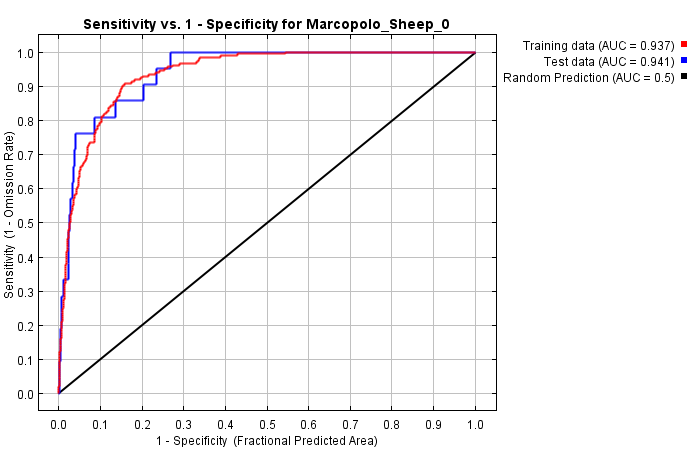

Supplement: Supplementary file 1 [file animals-15-01907-s001.zip › plots/Marcopolo_Sheep_0_roc.png]

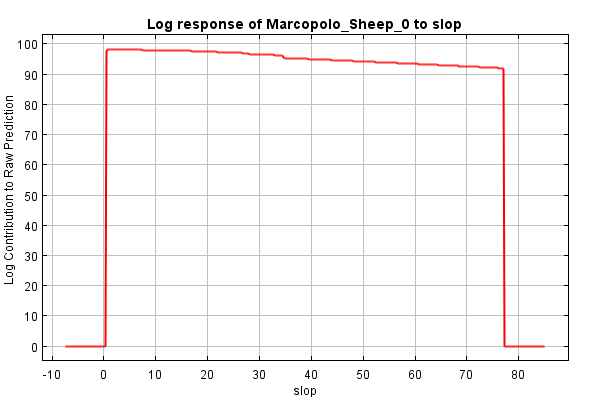

Supplement: Supplementary file 1 [file animals-15-01907-s001.zip › plots/Marcopolo_Sheep_0_slop.png]

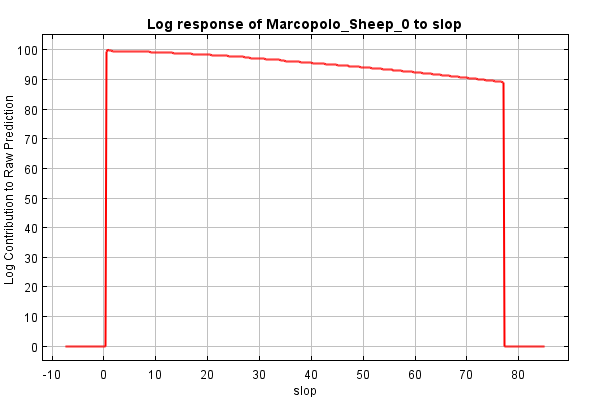

Supplement: Supplementary file 1 [file animals-15-01907-s001.zip › plots/Marcopolo_Sheep_0_slop_only.png]

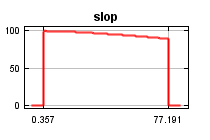

Supplement: Supplementary file 1 [file animals-15-01907-s001.zip › plots/Marcopolo_Sheep_0_slop_only_thumb.png]

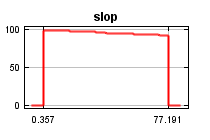

Supplement: Supplementary file 1 [file animals-15-01907-s001.zip › plots/Marcopolo_Sheep_0_slop_thumb.png]

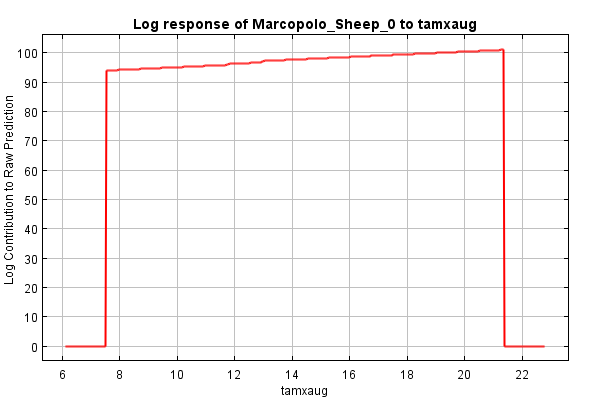

Supplement: Supplementary file 1 [file animals-15-01907-s001.zip › plots/Marcopolo_Sheep_0_tamxaug.png]

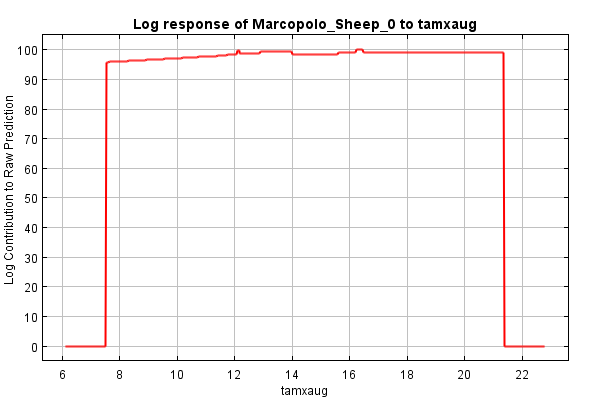

Supplement: Supplementary file 1 [file animals-15-01907-s001.zip › plots/Marcopolo_Sheep_0_tamxaug_only.png]

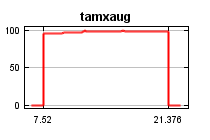

Supplement: Supplementary file 1 [file animals-15-01907-s001.zip › plots/Marcopolo_Sheep_0_tamxaug_only_thumb.png]

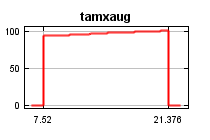

Supplement: Supplementary file 1 [file animals-15-01907-s001.zip › plots/Marcopolo_Sheep_0_tamxaug_thumb.png]

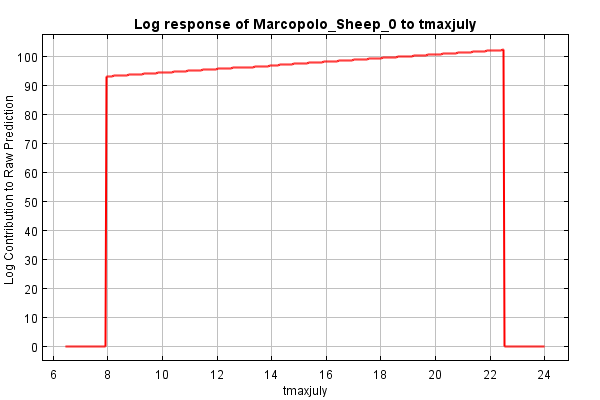

Supplement: Supplementary file 1 [file animals-15-01907-s001.zip › plots/Marcopolo_Sheep_0_tmaxjuly.png]

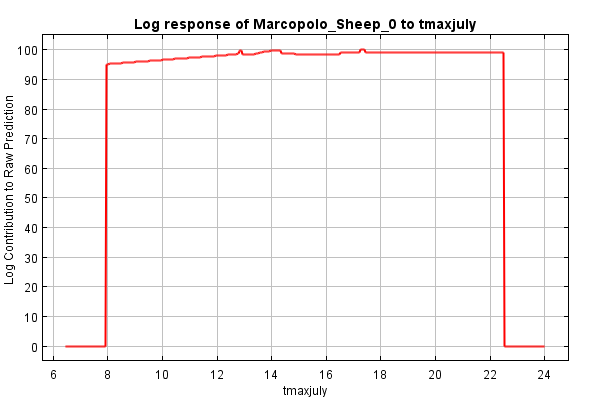

Supplement: Supplementary file 1 [file animals-15-01907-s001.zip › plots/Marcopolo_Sheep_0_tmaxjuly_only.png]

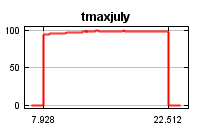

Supplement: Supplementary file 1 [file animals-15-01907-s001.zip › plots/Marcopolo_Sheep_0_tmaxjuly_only_thumb.png]

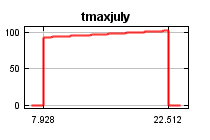

Supplement: Supplementary file 1 [file animals-15-01907-s001.zip › plots/Marcopolo_Sheep_0_tmaxjuly_thumb.png]

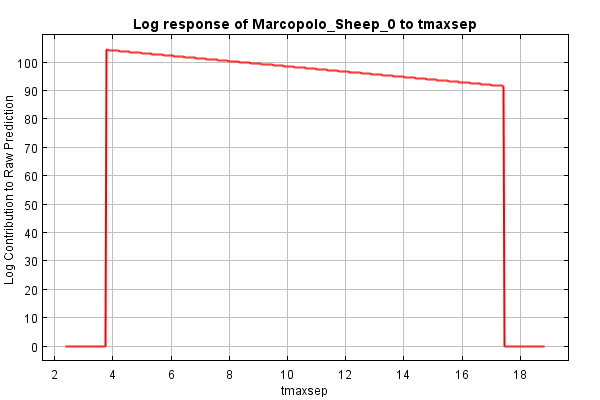

Supplement: Supplementary file 1 [file animals-15-01907-s001.zip › plots/Marcopolo_Sheep_0_tmaxsep.png]

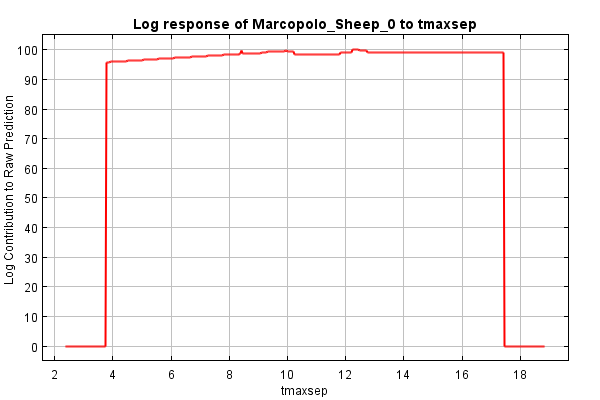

Supplement: Supplementary file 1 [file animals-15-01907-s001.zip › plots/Marcopolo_Sheep_0_tmaxsep_only.png]

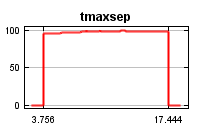

Supplement: Supplementary file 1 [file animals-15-01907-s001.zip › plots/Marcopolo_Sheep_0_tmaxsep_only_thumb.png]

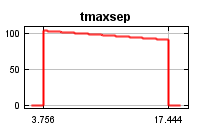

Supplement: Supplementary file 1 [file animals-15-01907-s001.zip › plots/Marcopolo_Sheep_0_tmaxsep_thumb.png]

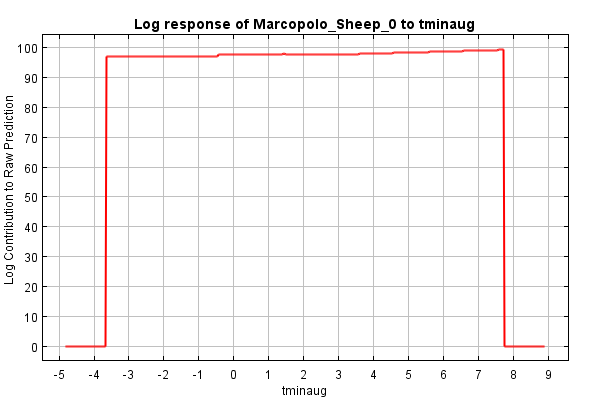

Supplement: Supplementary file 1 [file animals-15-01907-s001.zip › plots/Marcopolo_Sheep_0_tminaug.png]

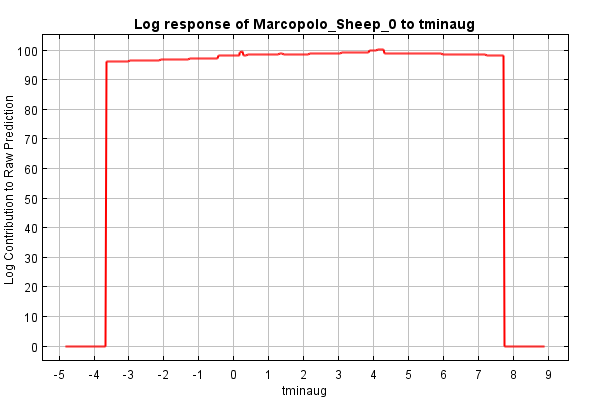

Supplement: Supplementary file 1 [file animals-15-01907-s001.zip › plots/Marcopolo_Sheep_0_tminaug_only.png]

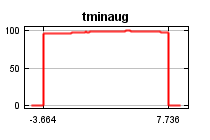

Supplement: Supplementary file 1 [file animals-15-01907-s001.zip › plots/Marcopolo_Sheep_0_tminaug_only_thumb.png]

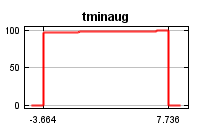

Supplement: Supplementary file 1 [file animals-15-01907-s001.zip › plots/Marcopolo_Sheep_0_tminaug_thumb.png]

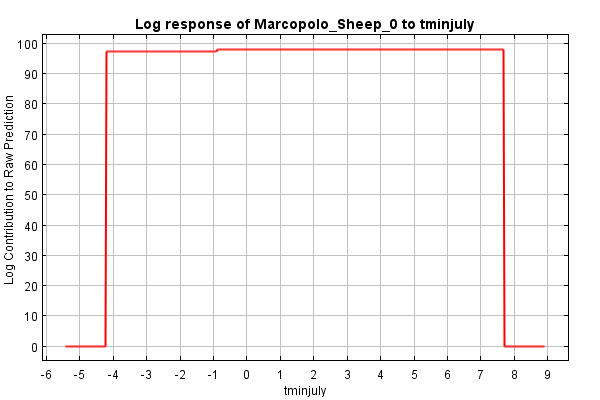

Supplement: Supplementary file 1 [file animals-15-01907-s001.zip › plots/Marcopolo_Sheep_0_tminjuly.png]

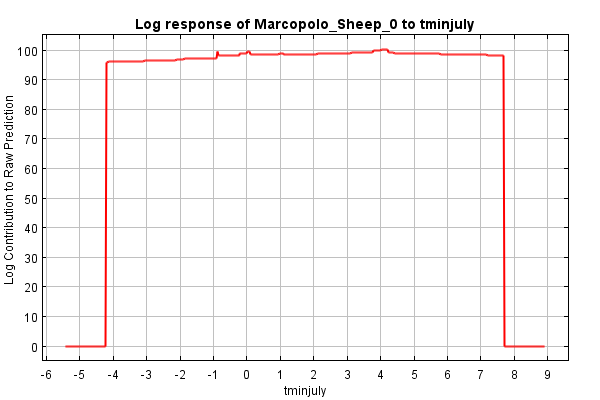

Supplement: Supplementary file 1 [file animals-15-01907-s001.zip › plots/Marcopolo_Sheep_0_tminjuly_only.png]

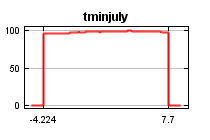

Supplement: Supplementary file 1 [file animals-15-01907-s001.zip › plots/Marcopolo_Sheep_0_tminjuly_only_thumb.png]

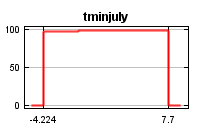

Supplement: Supplementary file 1 [file animals-15-01907-s001.zip › plots/Marcopolo_Sheep_0_tminjuly_thumb.png]

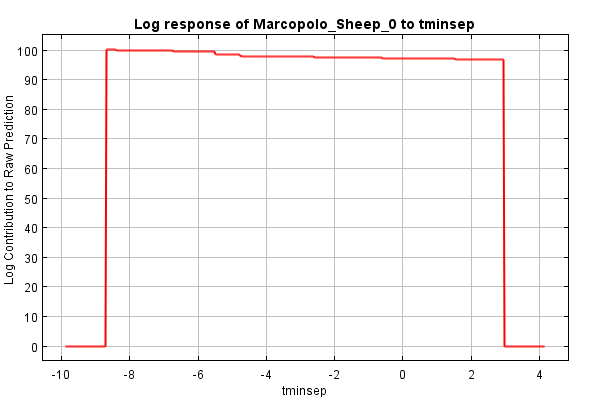

Supplement: Supplementary file 1 [file animals-15-01907-s001.zip › plots/Marcopolo_Sheep_0_tminsep.png]

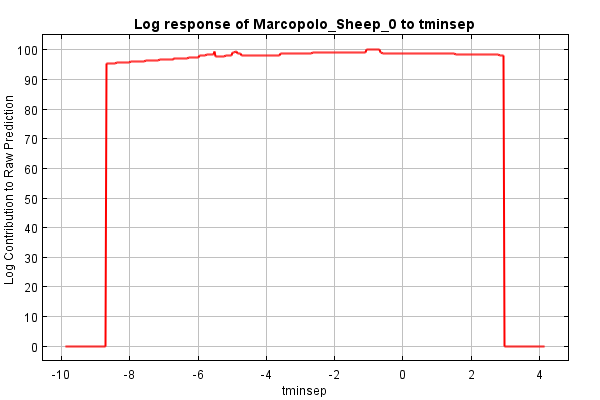

Supplement: Supplementary file 1 [file animals-15-01907-s001.zip › plots/Marcopolo_Sheep_0_tminsep_only.png]

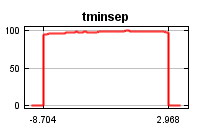

Supplement: Supplementary file 1 [file animals-15-01907-s001.zip › plots/Marcopolo_Sheep_0_tminsep_only_thumb.png]

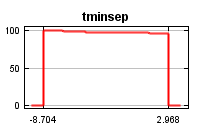

Supplement: Supplementary file 1 [file animals-15-01907-s001.zip › plots/Marcopolo_Sheep_0_tminsep_thumb.png]

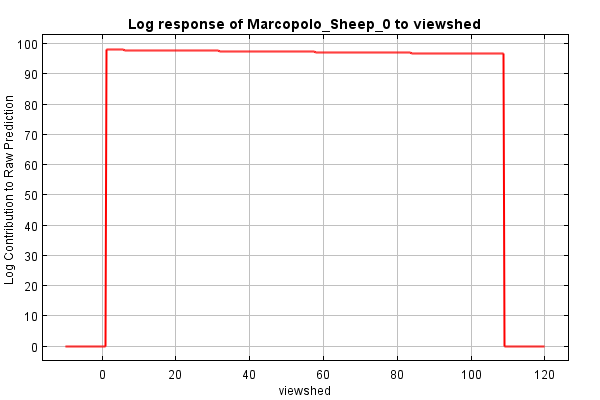

Supplement: Supplementary file 1 [file animals-15-01907-s001.zip › plots/Marcopolo_Sheep_0_viewshed.png]

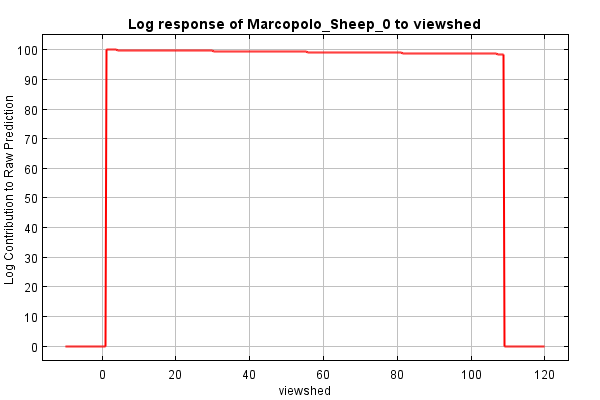

Supplement: Supplementary file 1 [file animals-15-01907-s001.zip › plots/Marcopolo_Sheep_0_viewshed_only.png]

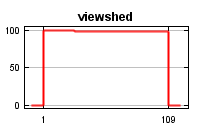

Supplement: Supplementary file 1 [file animals-15-01907-s001.zip › plots/Marcopolo_Sheep_0_viewshed_only_thumb.png]

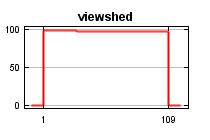

Supplement: Supplementary file 1 [file animals-15-01907-s001.zip › plots/Marcopolo_Sheep_0_viewshed_thumb.png]

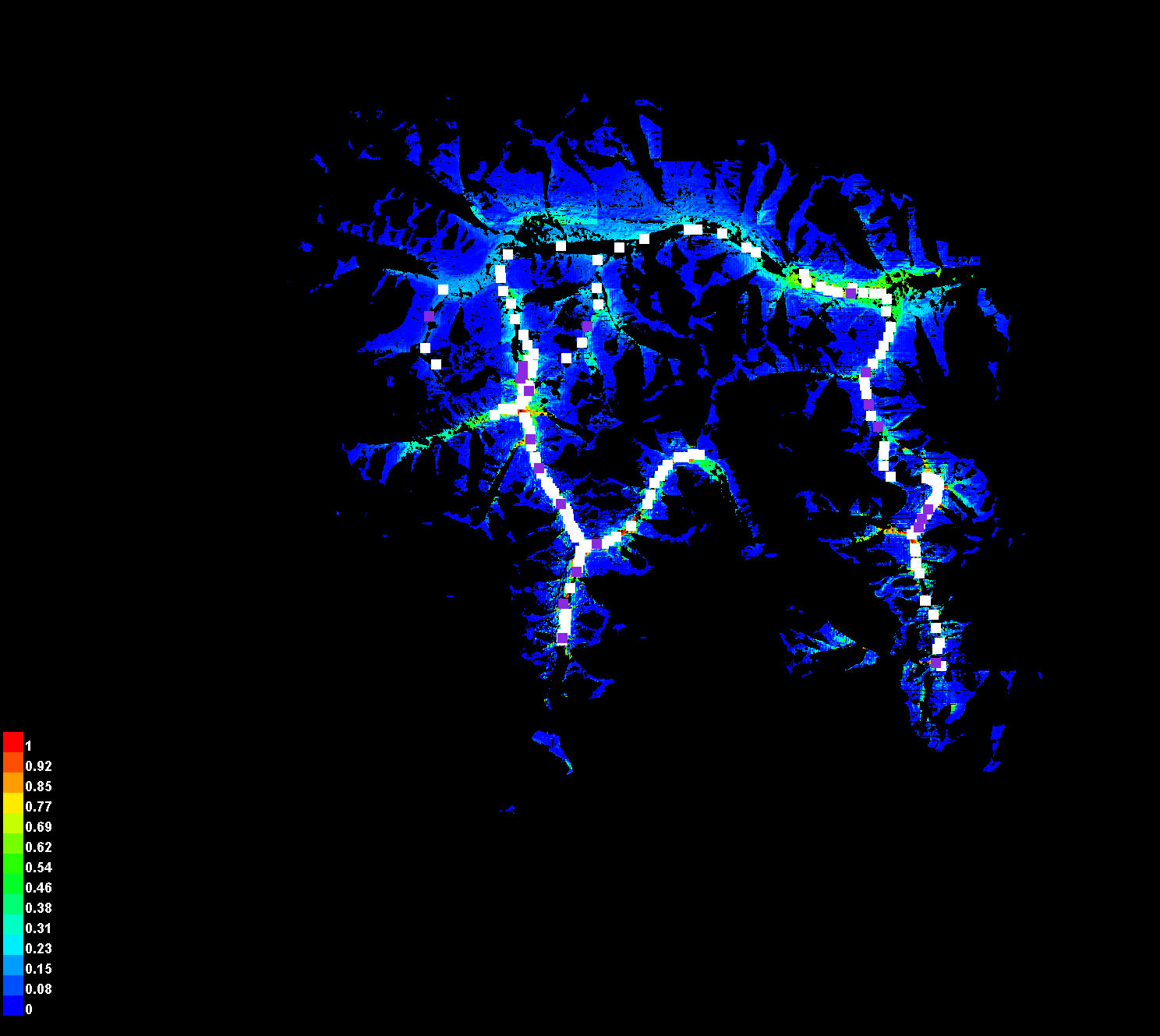

Supplement: Supplementary file 1 [file animals-15-01907-s001.zip › plots/Marcopolo_Sheep_1.png]

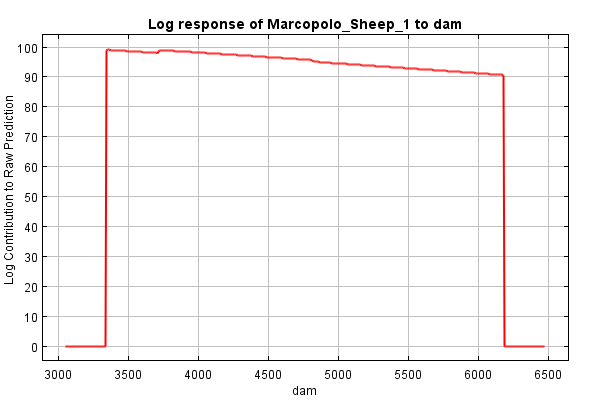

Supplement: Supplementary file 1 [file animals-15-01907-s001.zip › plots/Marcopolo_Sheep_1_dam.png]

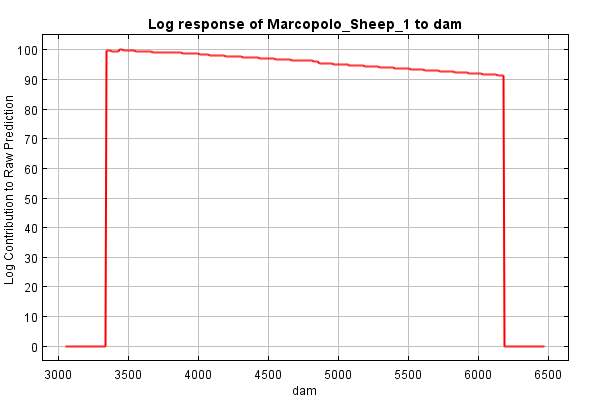

Supplement: Supplementary file 1 [file animals-15-01907-s001.zip › plots/Marcopolo_Sheep_1_dam_only.png]

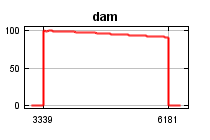

Supplement: Supplementary file 1 [file animals-15-01907-s001.zip › plots/Marcopolo_Sheep_1_dam_only_thumb.png]

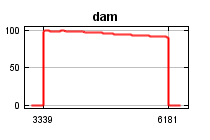

Supplement: Supplementary file 1 [file animals-15-01907-s001.zip › plots/Marcopolo_Sheep_1_dam_thumb.png]

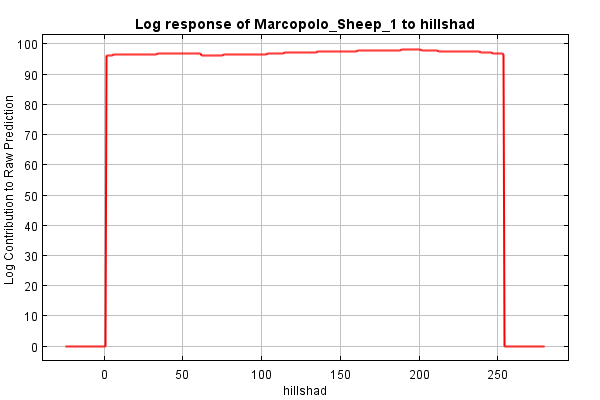

Supplement: Supplementary file 1 [file animals-15-01907-s001.zip › plots/Marcopolo_Sheep_1_hillshad.png]

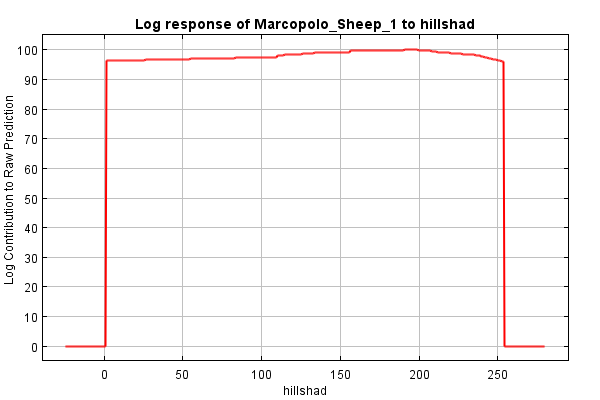

Supplement: Supplementary file 1 [file animals-15-01907-s001.zip › plots/Marcopolo_Sheep_1_hillshad_only.png]

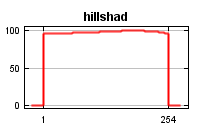

Supplement: Supplementary file 1 [file animals-15-01907-s001.zip › plots/Marcopolo_Sheep_1_hillshad_only_thumb.png]

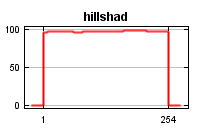

Supplement: Supplementary file 1 [file animals-15-01907-s001.zip › plots/Marcopolo_Sheep_1_hillshad_thumb.png]

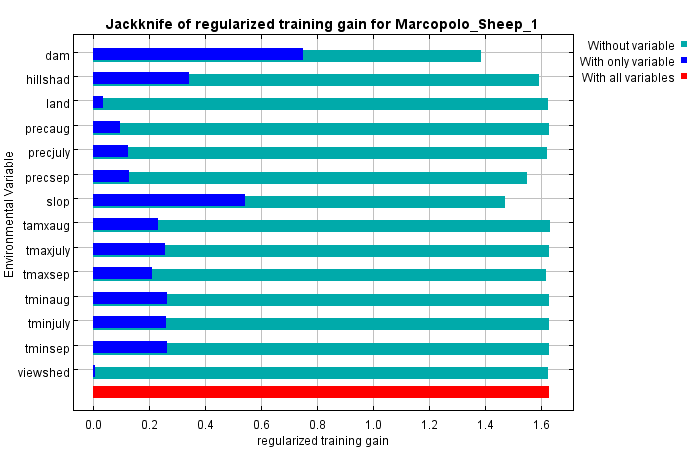

Supplement: Supplementary file 1 [file animals-15-01907-s001.zip › plots/Marcopolo_Sheep_1_jacknife.png]

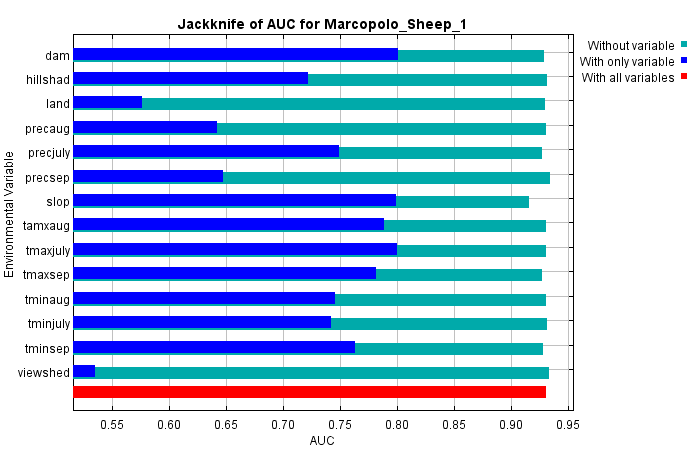

Supplement: Supplementary file 1 [file animals-15-01907-s001.zip › plots/Marcopolo_Sheep_1_jacknife_auc.png]

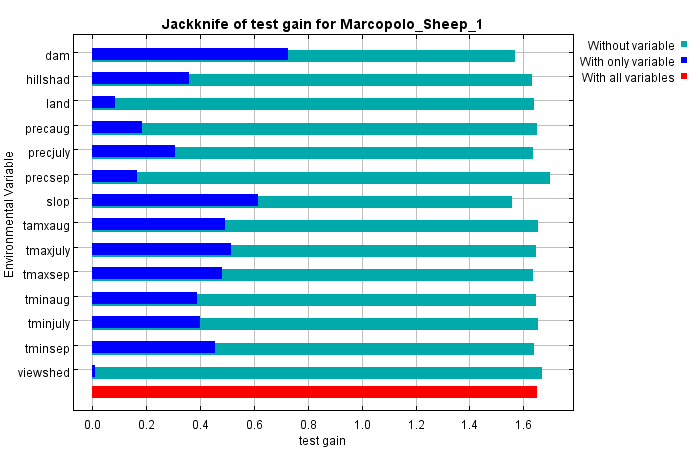

Supplement: Supplementary file 1 [file animals-15-01907-s001.zip › plots/Marcopolo_Sheep_1_jacknife_test.png]

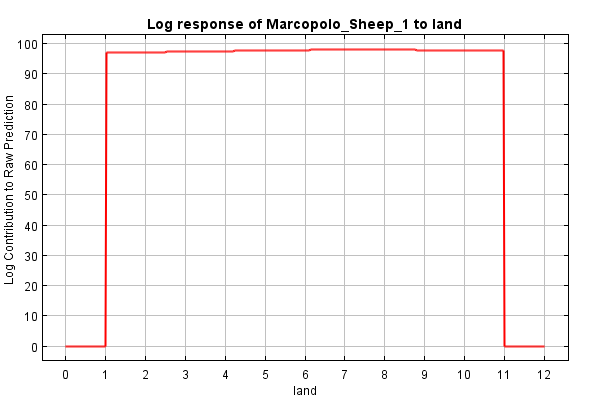

Supplement: Supplementary file 1 [file animals-15-01907-s001.zip › plots/Marcopolo_Sheep_1_land.png]

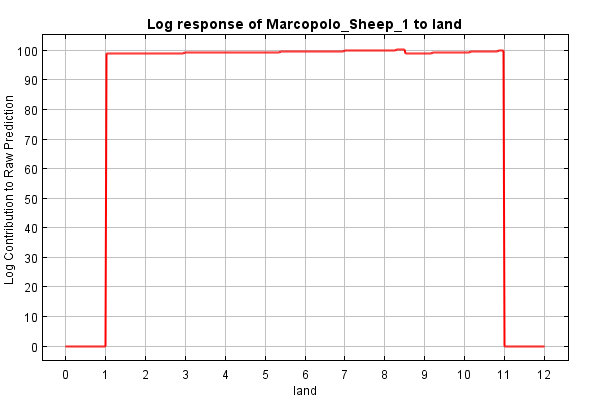

Supplement: Supplementary file 1 [file animals-15-01907-s001.zip › plots/Marcopolo_Sheep_1_land_only.png]

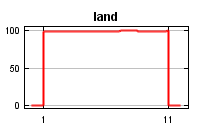

Supplement: Supplementary file 1 [file animals-15-01907-s001.zip › plots/Marcopolo_Sheep_1_land_only_thumb.png]

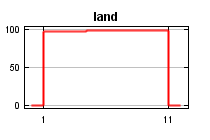

Supplement: Supplementary file 1 [file animals-15-01907-s001.zip › plots/Marcopolo_Sheep_1_land_thumb.png]

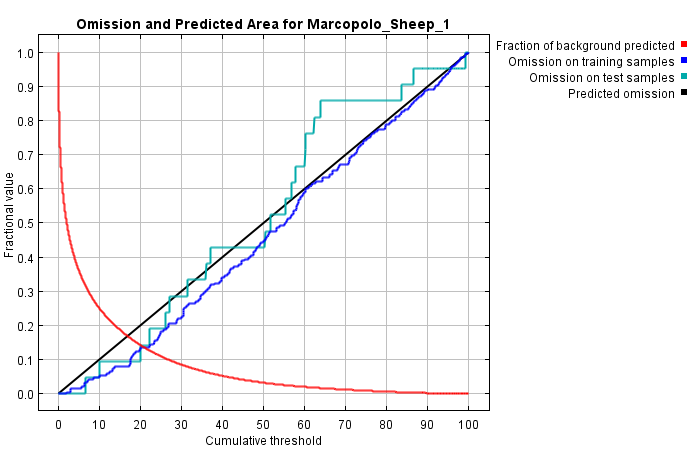

Supplement: Supplementary file 1 [file animals-15-01907-s001.zip › plots/Marcopolo_Sheep_1_omission.png]

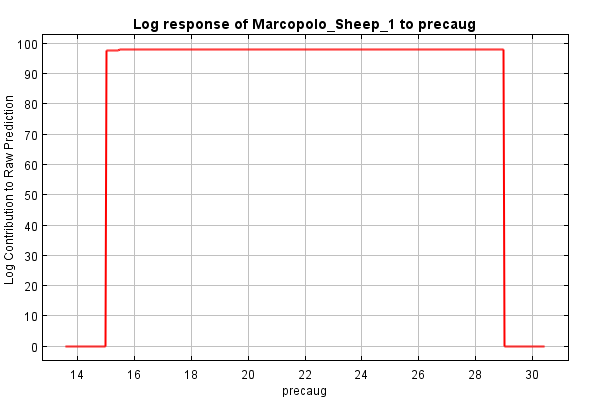

Supplement: Supplementary file 1 [file animals-15-01907-s001.zip › plots/Marcopolo_Sheep_1_precaug.png]

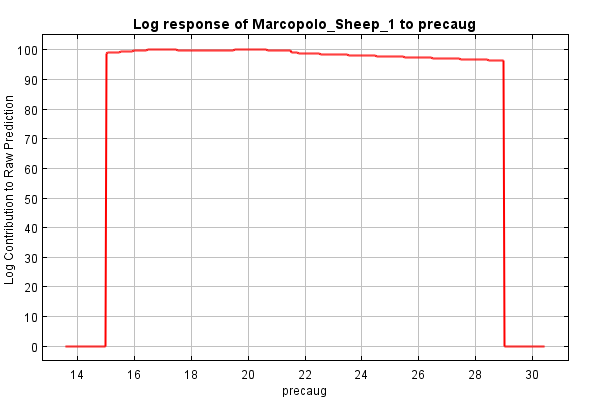

Supplement: Supplementary file 1 [file animals-15-01907-s001.zip › plots/Marcopolo_Sheep_1_precaug_only.png]

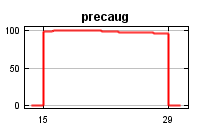

Supplement: Supplementary file 1 [file animals-15-01907-s001.zip › plots/Marcopolo_Sheep_1_precaug_only_thumb.png]

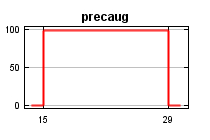

Supplement: Supplementary file 1 [file animals-15-01907-s001.zip › plots/Marcopolo_Sheep_1_precaug_thumb.png]

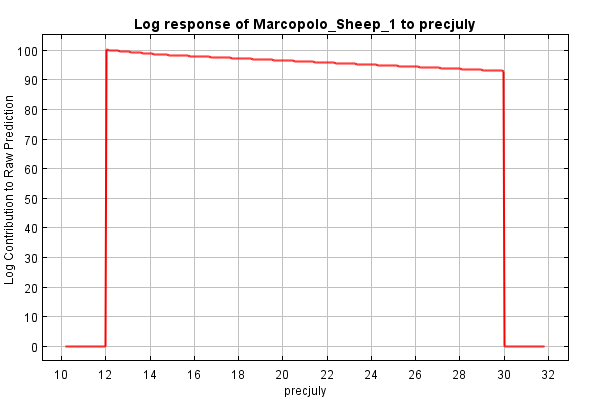

Supplement: Supplementary file 1 [file animals-15-01907-s001.zip › plots/Marcopolo_Sheep_1_precjuly.png]

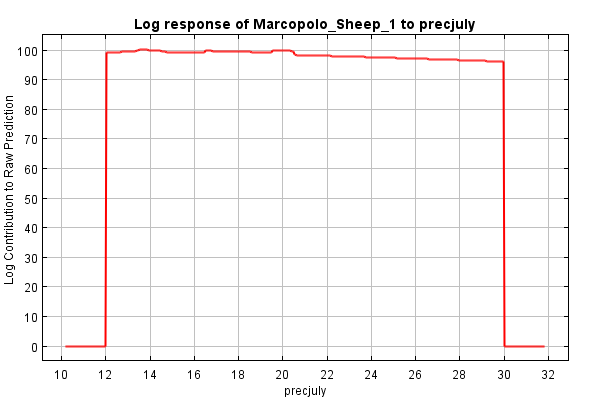

Supplement: Supplementary file 1 [file animals-15-01907-s001.zip › plots/Marcopolo_Sheep_1_precjuly_only.png]

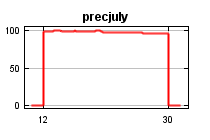

Supplement: Supplementary file 1 [file animals-15-01907-s001.zip › plots/Marcopolo_Sheep_1_precjuly_only_thumb.png]

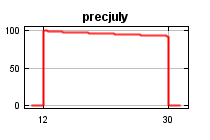

Supplement: Supplementary file 1 [file animals-15-01907-s001.zip › plots/Marcopolo_Sheep_1_precjuly_thumb.png]

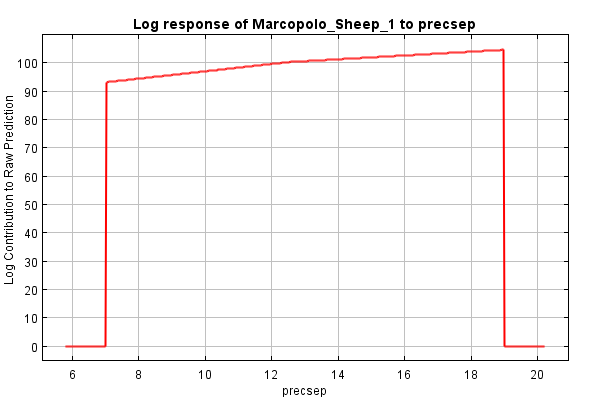

Supplement: Supplementary file 1 [file animals-15-01907-s001.zip › plots/Marcopolo_Sheep_1_precsep.png]

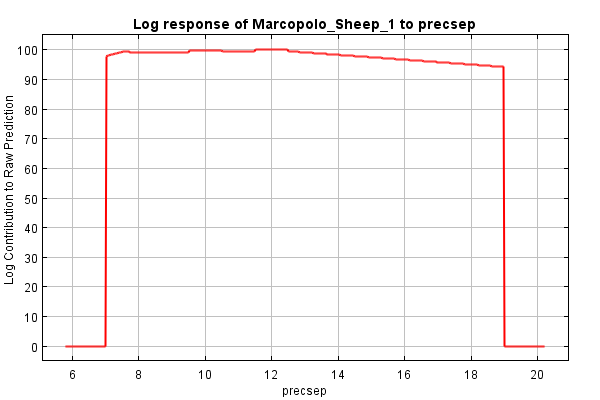

Supplement: Supplementary file 1 [file animals-15-01907-s001.zip › plots/Marcopolo_Sheep_1_precsep_only.png]

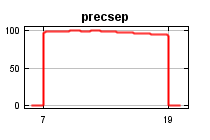

Supplement: Supplementary file 1 [file animals-15-01907-s001.zip › plots/Marcopolo_Sheep_1_precsep_only_thumb.png]

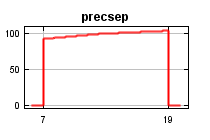

Supplement: Supplementary file 1 [file animals-15-01907-s001.zip › plots/Marcopolo_Sheep_1_precsep_thumb.png]

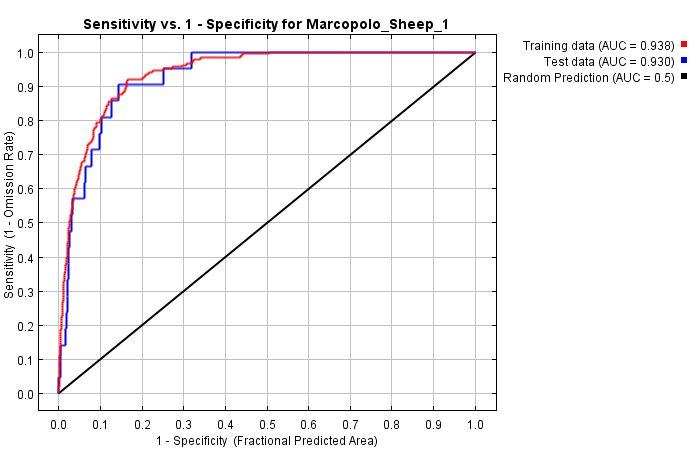

Supplement: Supplementary file 1 [file animals-15-01907-s001.zip › plots/Marcopolo_Sheep_1_roc.png]

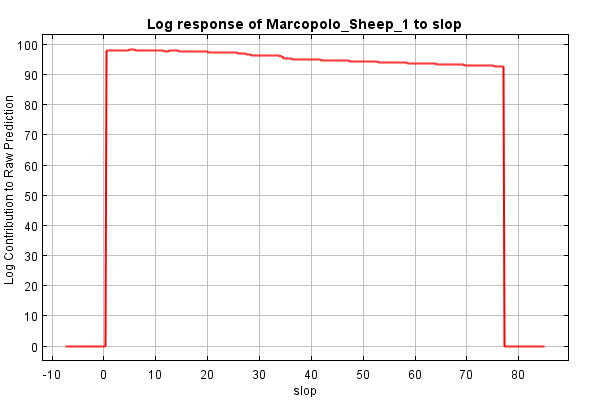

Supplement: Supplementary file 1 [file animals-15-01907-s001.zip › plots/Marcopolo_Sheep_1_slop.png]

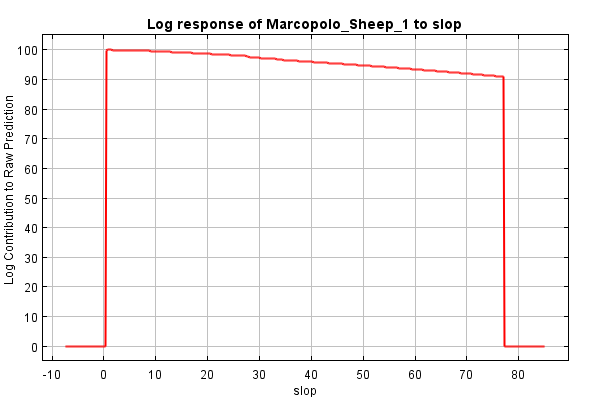

Supplement: Supplementary file 1 [file animals-15-01907-s001.zip › plots/Marcopolo_Sheep_1_slop_only.png]

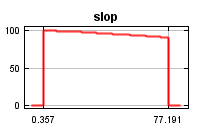

Supplement: Supplementary file 1 [file animals-15-01907-s001.zip › plots/Marcopolo_Sheep_1_slop_only_thumb.png]

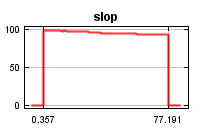

Supplement: Supplementary file 1 [file animals-15-01907-s001.zip › plots/Marcopolo_Sheep_1_slop_thumb.png]

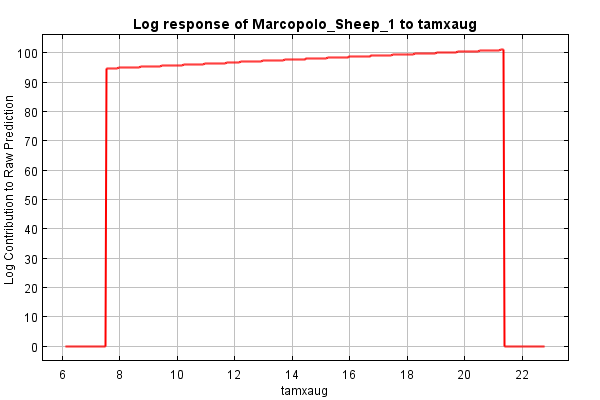

Supplement: Supplementary file 1 [file animals-15-01907-s001.zip › plots/Marcopolo_Sheep_1_tamxaug.png]

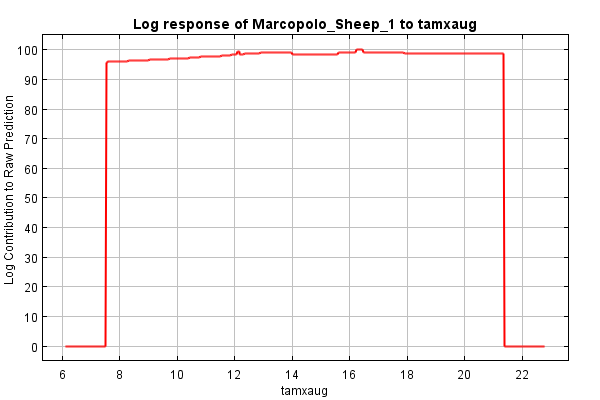

Supplement: Supplementary file 1 [file animals-15-01907-s001.zip › plots/Marcopolo_Sheep_1_tamxaug_only.png]

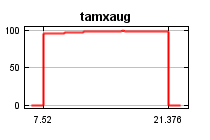

Supplement: Supplementary file 1 [file animals-15-01907-s001.zip › plots/Marcopolo_Sheep_1_tamxaug_only_thumb.png]

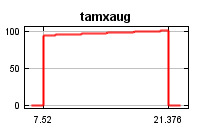

Supplement: Supplementary file 1 [file animals-15-01907-s001.zip › plots/Marcopolo_Sheep_1_tamxaug_thumb.png]
